# 3A and 2B proteins of SVA play chess game with host restriction factor DDX23 by apoptotic pathway

**DOI:** 10.1128/jvi.00761-25

**Published:** 2025-09-16

**Authors:** Jie Li, Haicheng Lin, Yi Zhou, Zongheng Lei, Xuan Wang, Ruimin Bi, Xuelan Liu, Jun Wang, Hongyao Zhang, Xiangxiang Wang, Jinsong Liu, Zongyi Bo, Haixiao Shen, Junfang Yan, Rui Tong, Yuting Xue, Minghao Zhuansun, Jinchi Zhou, Xinru Suo, Xinyue Chang, Zongjun Yin, Pei Sun, Liang Li

**Affiliations:** 1 College of Animal Science and Technology, Anhui Agricultural University 605541 , Hefei, Anhui, China; 2 Anhui Provincial Center for Disease Control and Prevention 117844, Hefei, Anhui, China; 3 Donghai County Bureau of Agriculture and Rural Affairs, Lianyungang, Jiangsu, China; 4 Joint International Research Laboratory of Agriculture and Agri-Product Safety, The Ministry of Education of China, Yangzhou University 38043, Yangzhou, China; 5 College of Veterinary Medicine, Nanjing Agricultural University 261674, Nanjing, China; 6 Key Laboratory of Applied Technology on Green-Eco-Healthy Animal Husbandry of Zhejiang Province, Zhejiang Provincial Engineering Laboratory for Animal Health Inspection and Internet Technology, College of Animal Science and Technology, College of Veterinary Medicine, Zhejiang A&F University 12627, Zhejiang, China; 7 Joint Research Center for Food Nutrition and Health of IHM, Anhui Agricultural University 12486, Hefei, People Republic of China; Loyola University Chicago - Health Sciences Campus, Maywood, Illinois, USA

**Keywords:** SVA, DDX23, 3A, 2B, caspase

## Abstract

Senecavirus A (SVA) is an emerging porcine pathogen that poses a significant threat to the global swine industry and has become an obstacle to its sustainable development. DEAD-box helicase 23 (DDX23), an RNA helicase, is increasingly recognized for its critical role in host antiviral defense mechanisms. However, the precise function of DDX23 in SVA infection remains unclear. In this study, we demonstrated through overexpression and knockout experiments that DDX23 inhibits SVA activity. Notably, compared to uninfected controls, SVA-infected BHK-21 cells exhibited a significant increase in DDX23 transcription levels but a decrease in DDX23 protein levels. Further co-transfection and inhibitor experiments revealed that DDX23 specifically targets leucine 14 (L14) of the SVA-3A protein and degrades SVA-3A protein via the Caspase-2/-6 pathway, thereby suppressing viral replication. Additionally, we identified that tryptophan (W44) and proline (P45) residues at positions 44 and 45 of SVA-2B are critical sites responsible for the reduced expression of DDX23 protein. This occurs through the Caspase-2/-3 pathway, leading to DDX23 degradation. Using reverse genetics, we constructed recombinant viruses and confirmed that K14 of SVA-3A and W44/P45 of SVA-2B are key regulatory amino acids for viral activity. These findings provide new insights into the mechanism by which DDX23 restricts SVA replication and how SVA evades host antiviral defenses, offering potential targets for future antiviral drug development and vaccine design.

**IMPORTANCE:**

The emergence and spread of Senecavirus A (SVA) have affected the healthy development of the global pig industry. In order to develop the pig industry, it is needed to explore the pathogenic mechanism of SVA and the strategies to evade the host antiviral defense. Our study is the first to elucidate the dual mechanisms by which DEAD-box helicase 23 (DDX23) inhibits SVA replication and how SVA counteracts DDX23 to promote its proliferation. By identifying key amino acid residues involved in these interactions, our findings provide a foundation for the development of targeted antiviral therapies and vaccines against SVA. These results have important practical value in alleviating the pressure of SVA on the economic and health development of the pig industry.

## INTRODUCTION

Seneca virus disease is an infectious disease in animals caused by Senecavirus A (SVA), a member of the small RNA virus family (1). SVA is the only species in the Seneca Valley Virus (SVV) genus of the family, where SVA is relatively closely related to the member of the genus *Cardiovirus* (2–4). Morphologically, SVA virus particles exhibit a spherical, icosahedral symmetry with a diameter of 25–30 nm. These particles lack an envelope and are composed of 60 subunits formed by three to four polypeptides (3, 5). Genetically, the SVA genome is approximately 7.3 kb in length and encodes a single polyprotein, designated L-4-3-4, which undergoes proteolytic cleavage by viral proteases (2A and 3C) and host proteases to yield 11 mature proteins. These include four structural proteins (VP1, VP2, VP3, and VP4) and seven non-structural proteins (2A, 2B, 2C, 3A, 3B, 3C, and 3D) (2, 5). Clinically, SVA is recognized as a novel pathogen that primarily infects pigs. Its clinical manifestations resemble those of other vesicular diseases, with initial vesicles forming on the hooves, nasal mucosa, and tongue of infected pigs. These lesions often progress to ulcers and lameness, significantly compromising the survival rate of neonatal piglets (6).

RNA helicases are integral to various aspects of cellular RNA metabolism, encompassing processes such as transcription, splicing, translation, microRNA (miRNA) biosynthesis, and RNA decay (7–10). Among these, the DEAD-box RNA helicase (DDX) family represents the largest and most evolutionarily conserved group of RNA helicases. This family is characterized by the presence of 12 conserved motifs, including the signature DEAD (Asp-Glu-Ala-Asp) motif, and is active across different biological stages (11). While DEAD-box proteins are recognized for their essential roles in RNA metabolism, emerging evidence highlights their involvement in antiviral defense mechanisms. Notable examples include retinoic acid-inducible gene I (RIG-I)/DDX58, DDX20, DDX21, DDX17, and DDX41, which have been shown to contribute significantly to host immune responses against viral infections (12–17).

DEAD-box helicase 23 (DDX23) is a member of the DEAD-box protein family and serves as a core component of the U5 small ribonucleoprotein, playing a critical role in RNA splicing (18, 19). In addition to RNA processing, DDX23 has many biological properties, including host nucleotide metabolism and tumor signal transduction (20–22). Recent studies have further expanded its functional repertoire, demonstrating that DDX23 is integral to the regulation of viral proliferation. This regulation occurs through its roles in innate immunity and interactions with viral nucleic acids or proteins (23–25). For instance, DDX23 has been shown to activate downstream innate immune signaling pathways during vesicular stomatitis virus (VSV) infection (25). Additionally, DDX23 is involved in the infection processes of various viruses, including foot-and-mouth disease virus (FMDV), dengue virus, HIV, chronic hepatitis B virus, and astrovirus (23, 24, 26–28). Despite these findings, it remains unclear whether DDX23 can regulate the proliferation of SVA. Similarly, numerous studies have reported on the mechanisms by which viruses antagonize antiviral proteins to evade host immunity. For example, the SVV proteinase (3Cpro) inhibited interferon (IFN)-α signaling by degrading signal transducer and activator of transcription (STAT) 1, STAT2, and interferon regulatory factor 9 through its protease activity, which is dependent on the caspase pathway (29). In a related mechanism, the 1–48 and 100–128 amino acid regions of SVA-2B reduce the expression of antiviral signaling proteins, such as mitochondrial antiviral signaling proteins (MAVS), through interactions with Caspase-3/-9 proteins. This suppression ultimately impairs the production of type I IFN, further enabling the virus to evade host immune responses (30). Furthermore, studies have shown that DDX21 was an effective antiviral factor for suppressing SVA infection, and SVA 2B and 3C proteins induce the degradation of DDX21 via caspase to antagonize the antiviral activity of DDX21 (31). These findings highlight the complex interactions between viral proteins and host antiviral defenses; however, the molecular mechanism of whether DDX23 and other viral proteins of SVA are regulated by cysteine asparaginase to cause apoptosis is unknown and needs to be further explored.

Previous studies have reported that DDX proteins may play a regulatory role in SVA replication (31). On this basis, our research focuses on revealing that DDX23 can significantly inhibit SVA expression, and SVA, in turn, antagonizes DDX23 to promote virus proliferation. Further studies showed that DDX23 degraded SVA-3A through the Caspase-2/-6 pathway. DDX23 interacts with 3A. In contrast, SVA-2B degrades DDX23 via the Caspase-2/-3 pathway, and DDX23 also interacts with 2B. To investigate these interactions, we confirmed that K14 of SVA-3A and W44/P45 of SVA-2B are the key amino acids of DDX23 that regulate viral activity in SVA infection. We also successfully constructed mutant recombinant viruses of SVA-2B and SVA-3A using reverse genetic techniques and confirmed the roles of recombinant viral K14 of SVA-3A and W44/P45 of SVA-2B in viral proliferation. Taken together, these findings provide new insights into the interactions between SVA and host factors, with important implications for identifying antiviral targets and elucidating the mechanisms of SVA infection and pathogenicity.

## RESULTS

### Host protein DDX23 inhibited SVA, and endogenous DDX23 expression was abnormal after cell SVA infection

**Fig 1:**
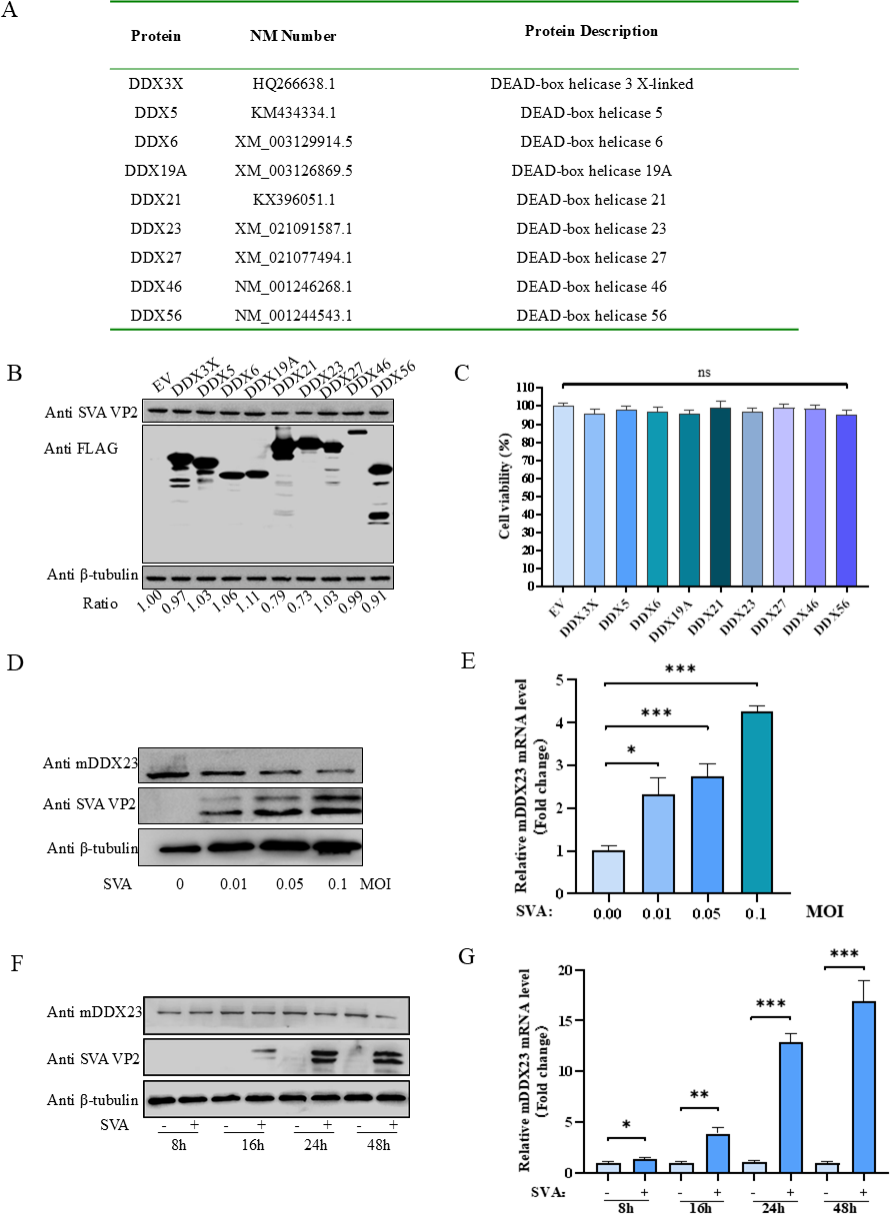
Host protein DDX23 inhibited SVA, and endogenous DDX23 expression was abnormal after cell SVA infection. (**A**) Porcine DDXs family screening list. (**B**) Western blot analysis of BHK-21 cells transfected with the specified gene after 18 h of infection with SVA. (**C**) Cell viability in cells overexpressing a specified gene. (**D and E**) BHK-21 cells infected with 0.01 MOI, 0.05 MOI, and 0.1 MOI SVA 24 h were collected for western blot and qRT-PCR to detect the protein level and transcription level of endogenous DDX23 in infected cells. (**F and G**) BHK-21 cells were infected with 0.1 MOI SVA for 8 h, 16 h, 24 h, and 48 h, and the BHK-21 cells were analyzed by western blot and qRT-PCR to detect the protein level and transcription level of endogenous DDX23. All the above western blot experiments used β-tubulin as the upper sample control. All samples run in triplicate. ***, *P* < 0.001; **, *P* < 0.01; *, *P* < 0.05; ns , *P* > 0.05.

Previous studies have shown that members of the DDX family exhibit antiviral properties. To explore this further, we randomly selected a subset of porcine DDX family genes (Fig. 1A). We conducted overexpression experiments on the selected subset of porcine DDX family genes in BHK-21 cells, in which DDX21 and DDX23 were identified as having inhibitory effects on the replication of SVA (Fig. 1B), and none of the DDX family genes in the subset were toxic to cells (Fig. 1C). In addition, we overexpressed DDX21 and DDX23 in BHK-21 cells, and the results showed a significant reduction in SVA virus titer and virus cDNA copy number (Fig. S1). Given that the antiviral role of DDX21 against SVA has been previously reported, we focused on DDX23 for subsequent investigations (31). Interestingly, we observed a notable phenomenon during these experiments. BHK-21 cells were inoculated with SVA at varying viral titers (0.01, 0.05, and 0.1 multiplicity of infection [MOI]). After incubation for 16 h, western blot results showed a dose-dependent decrease in DDX23 protein level (Fig. 1D), but quantitative real-time PCR (qRT-PCR) results showed a dose-dependent increase in DDX23 transcriptional activity after the same test operation (Fig. 1E). At 8 h, 16 h, 24 h, and 48 h after infection, protein samples and nucleic acid samples were collected to detect the amount of SVA virus, and the trend of the results was similar to the above experiment (Fig. 1F and G).

### After overexpression of DDX23, SVA was inhibited, and after knocking out DDX23, SVA proliferation increased

**Fig 2:**
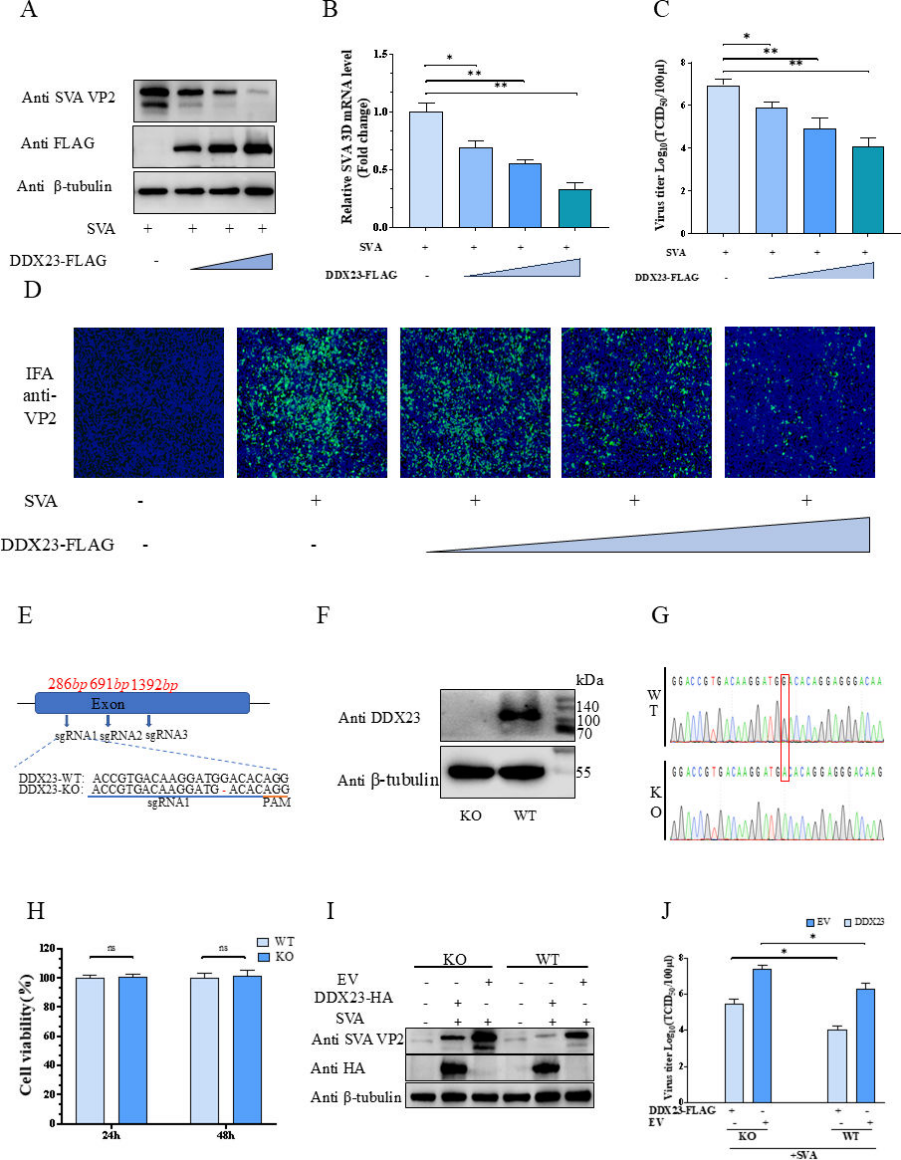
After overexpression of DDX23, SVA was inhibited, and after knocking out DDX23, SVA was proliferated. (**A**) BHK-21 cells transfected with pCA-DDX23-FLAG or EV were infected with SVA 18 h later, and cell lysate was collected 24 h later for western blot detection. VP2 and DDX23 were probed with anti-VP2 and anti-FLAG antibodies. (**B**) Quantitative detection of viral RNA levels by qRT-PCR. (**C**) The virus production in the supernatant of infected cells was detected by the TCID_50_ method. (**D**) IFA of SVA-infected cells. (**E**)single guide RNAs (sgRNAs) 1–3 targeting DDX23, AGG (yellow) is PAM. The highlighted base (red) indicates an indel. (**F**) Western blot analysis of DDX23 expression in BHK-DDX23-KO and BHK-WT cells. (**G**) Sanger sequencing showed DDX23 deletion. (**H**) Determination of BHK-DDX23-KO and BHK-WT cell viability. (**I**) pCA-DDX23-HA or EV was transfected into BHK-DDX23-KO and BHK-21 cells for 18 h and then infected with SVA. Samples were collected 24 h later for Western blot detection of viral titers in VP2 protein. (**J**) pCA-DDX23-FLAG or EV was transfected into BHK-DDX23-KO and BHK-21 cells for 18 h and then infected with SVA. Samples were collected 24 h later for TCID_50_ detection of viral titers in VP2 supernatant. All the above western blot experiments used β-tubulin as the upper sample control. All samples run in triplicate.

To further investigate the role of DDX23 in SVA replication, we transfected BHK-21 cells with gradient increased pCA-DDX23-FLAG or empty vector (EV) for 24 h and then infected cells with 0.01 MOI SVA for 18 h. We comprehensively evaluated virus proliferation using western blot, qRT-PCR, TCID50, and indirect immune fluorescence analysis (IFA) experiments. The results showed that in BHK-21 cells, with the increase of DDX23 expression level, the expression of SVA-VP2 protein, transcription of SVA-3D gene, and the titer of progeny virus in cell supernatant all showed a dose-dependent decrease (Fig. 2A through D). The above results confirm that the overexpression of DDX23 can effectively inhibit the proliferation of the SVA virus. In addition, we constructed DDX23 knockout BHK-21 cells (BHK-DDX23-KO) using CRISPR-Cas9 technology (Fig. 2E). Sequencing and western blot results confirmed that the DDX23 gene had been successfully knocked out in BHK-21 cells (Fig. 2F and G). Cell viability assay showed no difference between BHK-DDX23-KO and BHK-WT (Fig. 2H). Subsequently, the replication efficiency of SVA in BHK-DDX23-KO and BHK-WT cells was measured. Detection of VP2 protein levels and viral titers by western blot and TCID_50_ showed a significant increase in SVA replication levels in BHK-21 cells compared to wild type (WT). Overexpression of DDX23 in knockout cells inhibits SVA replication (Fig. 2I and J). These results indicate that DDX23 has no effect on viral binding and entry but significantly inhibits SVA replication.

### DDX23 exerts inhibitory effect during virus replication

**Fig 3:**
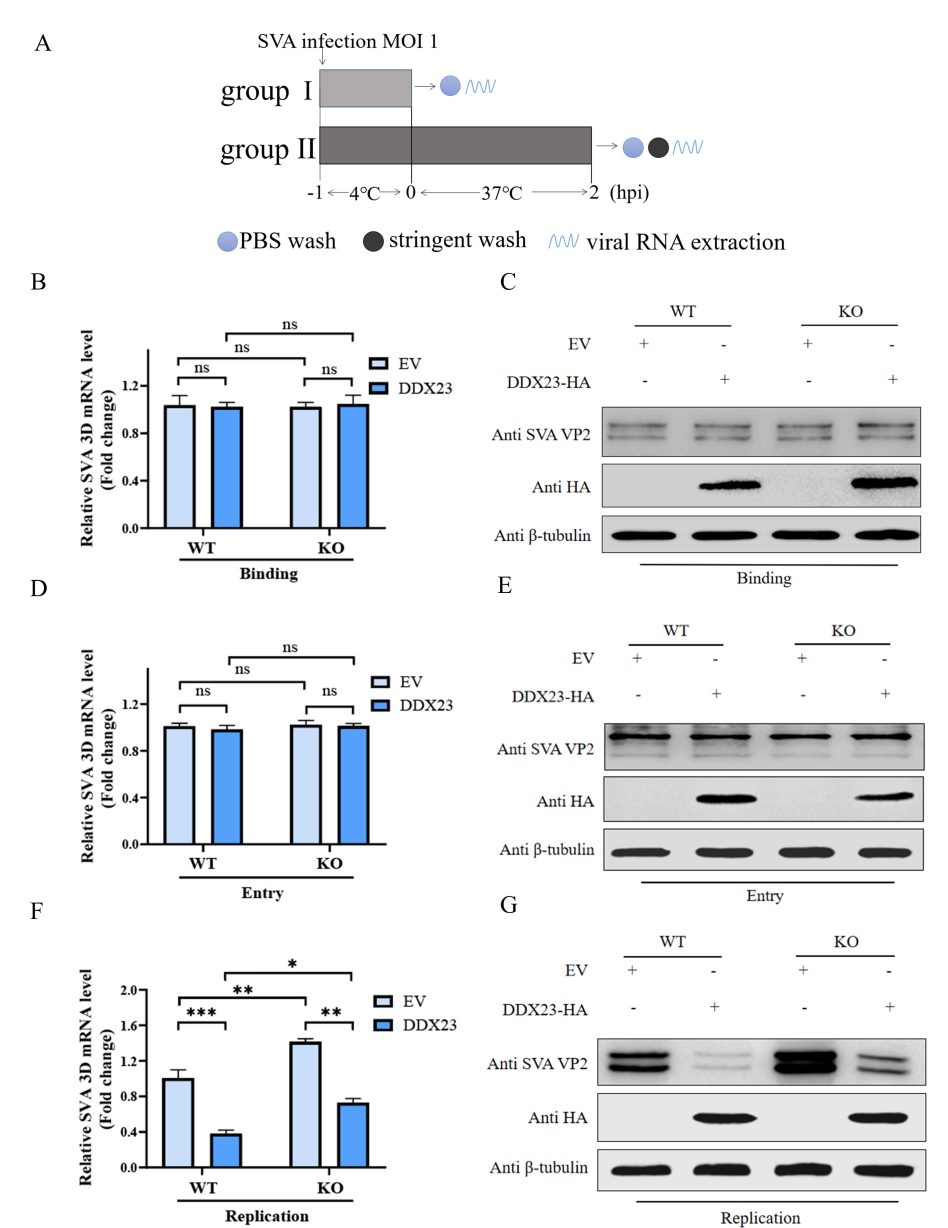
DDX23 exerts an inhibitory effect during virus replication. (**A**) Flow chart of the influence of DDX23 on SVA binding and entry. (**B and C**) Uniformly plate BHK-21 cells and BHK-DDX23-KO cells in 12-well plates, transfected with DDX23 or empty carrier, inoculated with 1 MOI SVA virus 24 h later, incubated at 4°C for 1 h, washed with PBS, and cell samples were collected. Western blotting and qRT-PCR were used to detect the changes in viral protein levels and transcription levels. (**D and E**) Uniformly plate BHK-21 cells and BHK-DDX23-KO cells in 12-well plates, transfected with DDX23 or empty carrier, inoculated with 1 MOI SVA virus 24 h later, incubated at 4°C for 1 h and washed with PBS, then replaced the culture medium and incubated at 37°C for 2 h, and finally washed with high-salt sodium citrate solution for 3 minutes to remove the virus that did not enter the cells. The cell samples were collected and detected by western blotting and qRT-PCR. (**F and G**) Uniformly plate BHK-21 cells and BHK-DDX23-KO cells in 12-well plates and transfect with DDX23 or EV, respectively. Post 24 h, SVA virus with 1 MOI was inoculated. Post 16 h, cell samples were collected, and the changes of the virus were detected by western blotting and qRT-PCR. All the above western blot experiments used β-tubulin as the upper sample control. All samples run in triplicate. *, *P* < 0.05; ns, P > 0.05.

To determine the specific stages of the SVA life cycle affected by DDX23, we conducted experiments in BHK-WT and BHK-DDX23-KO, respectively. We first investigated its effects on viral binding and entry phases (Fig. 3A). In the binding experiment, cells were incubated with SVA at an MOI of 1.0 for 1 h at 4°C, followed by washing with phosphate-buffered saline (PBS) to remove unbound virus. In the entry experiment, cells were similarly incubated at 4°C for 1 h but were subsequently shifted to 37°C for 2 h to allow viral entry. Afterward, the cells were washed with a high-salt solution to eliminate any non-internalized virus. Intracellular viral loads were then quantified using western blot and qRT-PCR. The results revealed no significant differences in viral quantities between DDX23-transfected and EV-transfected cells during the binding (Fig. 3B and C) or entry phases of SVA infection (Fig. 3D and E), regardless of whether experiments were conducted in BHK-WT or BHK-DDX23-KO. To further assess the role of DDX23 in viral replication, a third experiment was conducted. BHK-21 cells and BHK-DDX23-KO cells were transfected with DDX23 or EV, respectively, and then infected with SVA virus. They were cultured at 37°C for 6 h. Following three PBS washes to remove extracellular virus, intracellular viral loads were again measured via western blot and qRT-PCR. However, during the replication phase, the viral load in BHK-21 cells transfected with DDX23 was significantly lower than that in the EV-transfected control group. In the BHK-DDX23-KO group, there was a significant increase in SVA replication. However, with the replenishment of DDX23 transfection, SVA replication showed a certain degree of decrease (Fig. 3F and G). These results indicate that DDX23 has no effect on viral binding and entry but significantly inhibits SVA replication.

### DDX23 degrades SVA-3A protein and interacts with it

**Fig 4:**
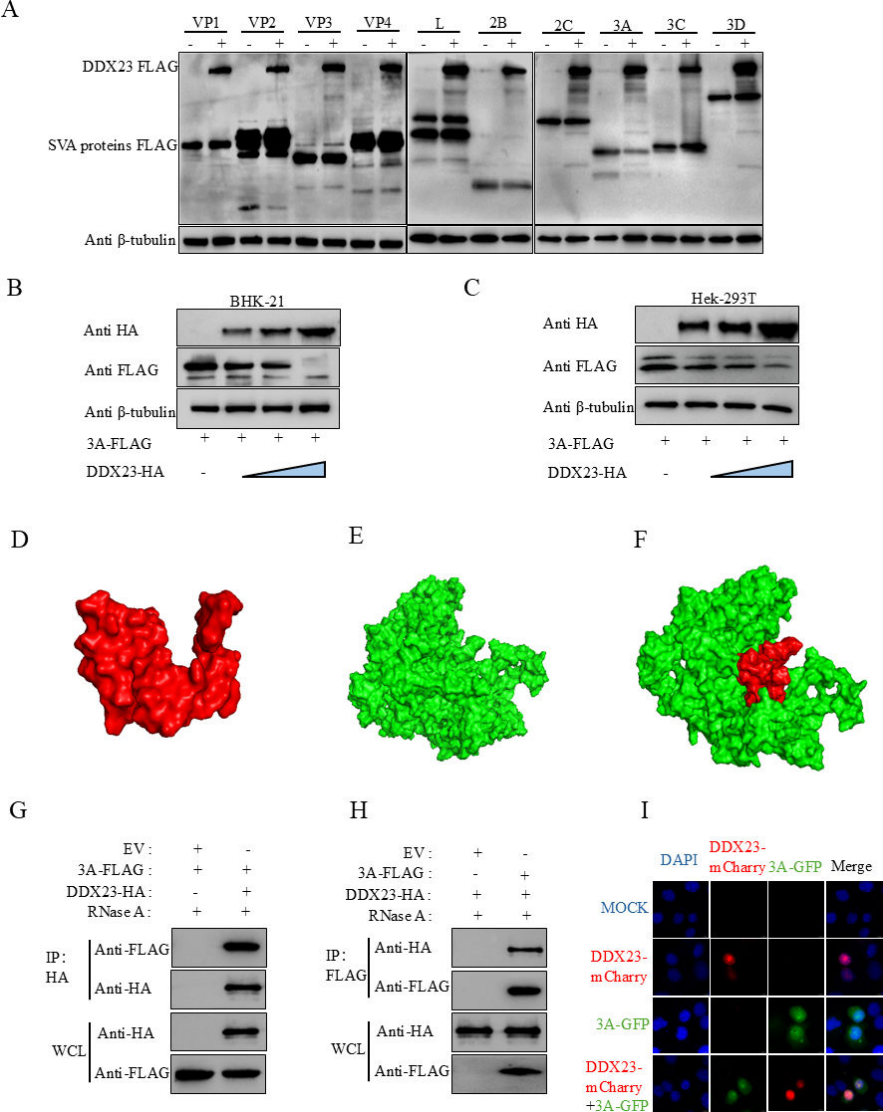
DDX23 degrades SVA-3A protein and interacts with it. (**A**) HEK-293T cells were transfected with the indicated plasmids. After incubating for 24 h, western blot assays were performed to evaluate the expression of pCA-DDX23-FLAG and FLAG-tagged viral proteins. (**B**) BHK-21 cells were transfected with 3A-FLAG along with HA-tagged vector or increasing quantities of pCA-DDX23-HA plasmid, and the protein expressions were evaluated by western blot. (**C**) HEK-293T cells were transfected with 3A-FLAG along with HA-tagged vector or increasing quantities of pCA-DDX23-HA plasma, and the protein expressions were evaluated by western blot. (**D**) 3A protein 3D model. (**E**) DDX23 3D model. (**F**) 3D results of predicted docking of DDX23 and SVA 3A proteins. (**G and H**) DDX23 with HA label and SVA-3A with FLAG label were transfected into HEK-293T cells for co-immunoprecipitation (Co-IP) test. (**I**) DDX23 was co-located with SVA-3A, and pmCherry-DDX23 (red light) and pEGFP-3A (green light) were co-transfected into BHK-21 cells and observed on a confocal laser microscope according to IFA test procedures. All the above western blot experiments used β-tubulin as the upper sample control. All samples run in triplicate.

To further investigate the mechanism by which DDX23 inhibits SVA infection, we examined the relationship between DDX23 and SVA proteins. Specifically, we co-transfected HEK-293T cells with either a DDX23-FLAG plasmid or an empty vector control, along with FLAG-tagged SVA viral proteins (L, VP1, VP2, VP3, VP4, 2B, 2C, 3A, 3B, 3C, and 3D). After 24 h, protein expression was analyzed via western blot. The results revealed that DDX23 significantly suppressed the expression of the 3A protein (Fig. 4A). To further assess this effect, BHK-21 and HEK-293T cells were transfected with varying doses of DDX23 plasmids and 3A plasmids for 24 h. Western blot analysis demonstrated that DDX23-mediated degradation of the 3A protein occurred in a dose-dependent manner (Fig. 4B and C).

Having established that DDX23 degrades SVA-3A, we next investigated whether DDX23 interacts with the 3A protein. The three-dimensional structures of DDX23 protein and SVA-3A were constructed by AlphaFold, and protein-protein molecular docking was performed using the ClusPro 2.0 protein docking web server. The protein three-dimensional structure and docking results were visualized by PyMol. Two indicators are included, one is the docking score, a more negative docking score indicates a more likely binding model; the other is the confidence score, when the confidence score is higher than 0.7, the possibility of two molecules binding is high; when the confidence score is between 0.5 and 0.7, it is considered that the two molecules can be bound; and when the confidence score is lower than 0.5, it is considered that the two molecules have a lower possibility of binding. The results after docking using HDOCK are shown in Fig. 4D, the model_1 with the lowest binding energy has a docking score of −211.10 and a confidence score of 0.7724. This indicates that the probability of protein-protein binding in this form is high. The simulation results indicated a high likelihood of interaction between the two proteins (Fig. 4D through F). To validate this prediction experimentally, we conducted a co-immunoprecipitation (Co-IP) assay. HEK-293T cells were co-transfected with HA-tagged DDX23 and FLAG-tagged SVA-3A eukaryotic expression plasmids. The Co-IP results confirmed that DDX23 physically interacts indirectly with the SVA-3A protein (Fig. 4G and H). Finally, to visualize the subcellular localization of these interactions, we constructed green fluorescent protein (GFP)-labeled 3A and mCherry-labeled DDX23 plasmids, transfected them into BHK-21 cells, and observed them with confocal laser microscopy. Confocal imaging results showed that DDX23 and SVA-3A were colocalized in the nucleus (Fig. 4I).

### DDX23 degraded SVA-3A through Caspase2 and Caspase6

**Fig 5:**
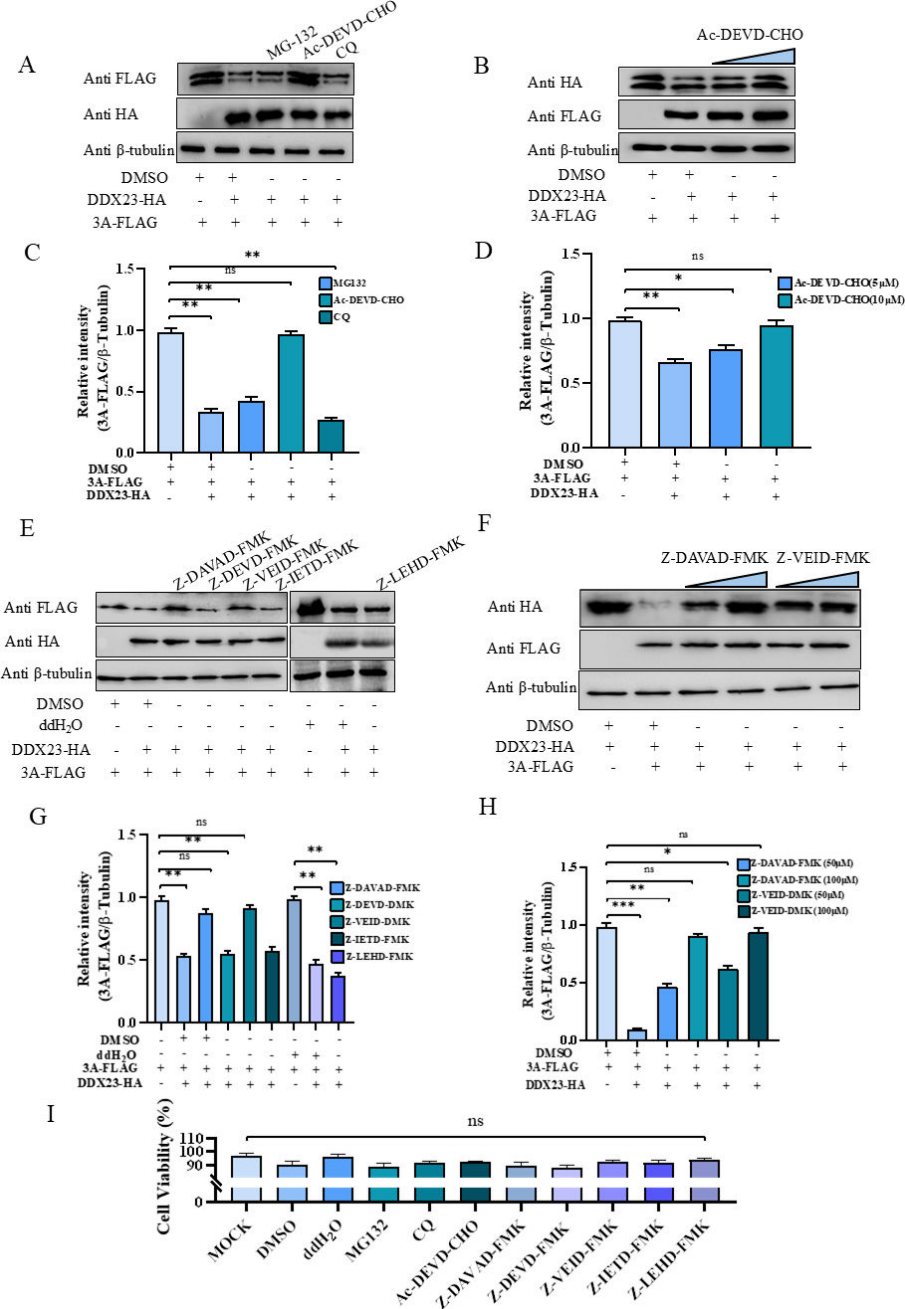
DDX23 degraded SVA-3A through Caspase3 and Caspase6. (**A and C**) pCA-3A-FLAG and pCA-DDX23-HA or EV were transfected into HEK-293T cells, and different inhibitors (MG132, CQ, or Ac-DEVD-CHO) were added 8 h later to affect the cells, while DMSO was set as a negative control group. Sixteen-hour cell samples were collected for western blot analysis to detect the changes in the 3A protein. 3A/β-tubulin protein levels are depicted as relative intensities. (**B and D**) Cells transfected with 3A and DDX23 were treated with different doses of Ac-DEVD-CHO, and cell samples were collected for western blot assay. 3A/β-tubulin protein levels are depicted as relative intensities. (**E and G**) HEK-293T cells were transfected with pCA-3A-FLAG, pCA-DDX23-HA, or EV, and treated with different inhibitors (Z-VDVAD-FMK, Z-DEVD-FMK, Z-VEID-FMK, Z-IETD-FMK, and Z-LEHD-FMK) after 8 h, while DMSO and ddH_2_O were set as a negative control group. The cell samples were collected 16 h later for western blot analysis to detect the changes in DDX23 protein. 3A/β-tubulin protein levels are depicted as relative intensities. (**F and H**) Cells transfected with 3A and DDX23 were treated with different doses of Z-VDVAD-FMK and Z-VEID-FMK, and cell samples were collected for western blot assay. 3A/β-tubulin protein levels are depicted as relative intensities. (**I**) Cell viability was measured after treatment with the above-mentioned inhibitors. The above western blot experiments used β-tubulin as the upper sample control. All samples run in triplicate.

In order to determine the pathway by which DDX23 degrades SVA-3A, plasmids pCA-DDX23-HA and pCA-3A-FLAG were co-transfected into BHK-21 cells. After transfection for 8 h, they were treated with MG-132, Ac-DEVD-CHO, and CQ for 16 h, respectively. In these treatments, 3A protein levels recovered with interference from Ac-DEVD-CHO, while MG-132 and CQ failed to restore 3A protein levels (Fig. 5A through C). Meanwhile, the recovery effect of 3A protein was dose-dependent with the dose of Ac-DEVD-CHO (Fig. 5B through D). To further elucidate the mechanism of action, we identified the degradation pathway of Caspase. Plasmids pCA-DDX23-HA and pCA-3A-FLAG were co-transfected into BHK-21 cells. After 8 h, cells were treated with specific inhibitors targeting Caspase-2/-3/-6/-8/-9 (Z-VDVAD-FMK, Z-DEVD-FMK, Z-VEID-FMK, Z-IETD-FMK, and Z-LEHD-FMK, respectively) for 16 h. Western blot analysis showed that 3A protein levels recovered when treated with Z-VDVAD-FMK and Z-VEID-FMK, while other inhibitors failed to restore 3A protein levels (Fig. 5E through G). Similarly, the recovery effect of 3A protein was dose-dependent with the Z-VDVAD-FMK and Z-VEID-FMK doses (Fig. 5F through H). These findings suggest that Caspase-2 and Caspase-6 play a key role in DDX23-induced 3A degradation. Importantly, a cell viability assay confirmed that the application of all inhibitors and control reagents (ddH_2_O and dimethyl sulfoxide [DMSO]) did not result in significant changes in cell growth or viability, ensuring that the observed effects were not due to cytotoxicity (Fig. 5I).

### DDX23 causes degradation of 3A by targeting its 14-position leucine residue

**Fig 6:**
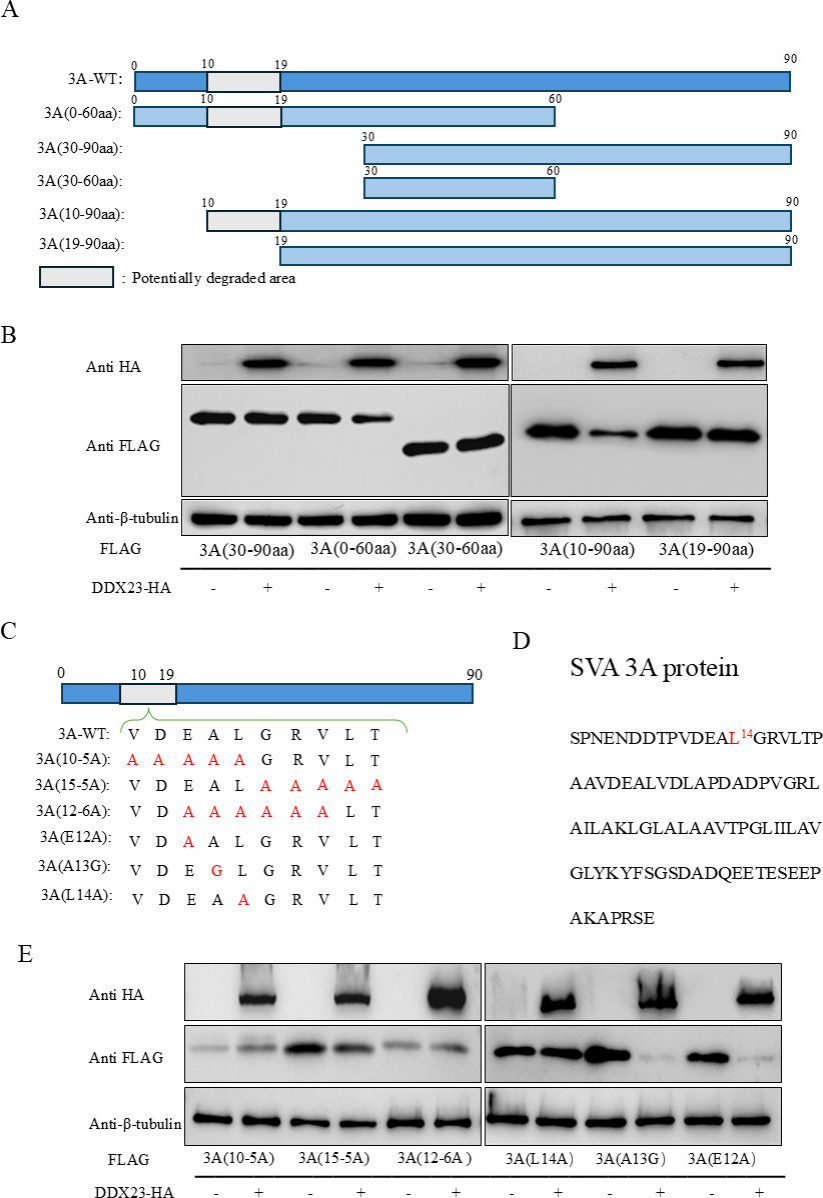
Leucine residue 14 of 3A is a key site for inducing degradation. (**A**) Construct SVA-3A truncation diagram. (**B**) HEK-293T cells were transfected with SVA-3A truncated body and plasmid pCA-DDX23-HA, and samples were collected 24 h later for western blot analysis. (**C**) Construct a schematic diagram of SVA-3A mutant. (**D**) Schematic diagram of amino acids at SVA-3A action site. (**E**) SVA-3A mutant and plasmid pCA-DDX23-HA were co-transfected into HEK-293T cells, and samples were collected 24 h later for western blot analysis. The above western blot experiments used β-tubulin as the upper sample control. All samples run in triplicate.

After determining the degradation of SVA-3A by DDX23 through the Caspase-2/-6 pathway, in order to further identify the determinant of 3A affected by DDX23, we constructed five 3A truncated expression plasmids with FLAG markers. The scope and naming of these truncations are 0–60 aa, 30–90 aa, 30–60 aa, 10–90 aa, and 19–90 aa (Fig. 6A). These plasmids were co-transfected with plasmid pCA-DDX23-HA into HEK-293T cells for 24 h, and cell lysates were collected for western blot (Fig. 6B). After screening, it was found that the key sites involved in the degradation of 3A were located at 10-20aa, and crucial residues of the 10–22 aa region were mapped using alanine scanning, and mutants were generated. The mutants were named 3A(10-5A), 3A(15-5A), 3A(12-6A), 3A(E12A), 3A(A13G), and 3A(L14A). The schematic diagram is shown in Fig. 6C. The results showed that leucine at position 14 of 3A was the key site involved in the degradation of DDX23 (Fig. 6D and E).

### SVA-2B antagonizes DDX23 and interacts with DDX23

**Fig 7:**
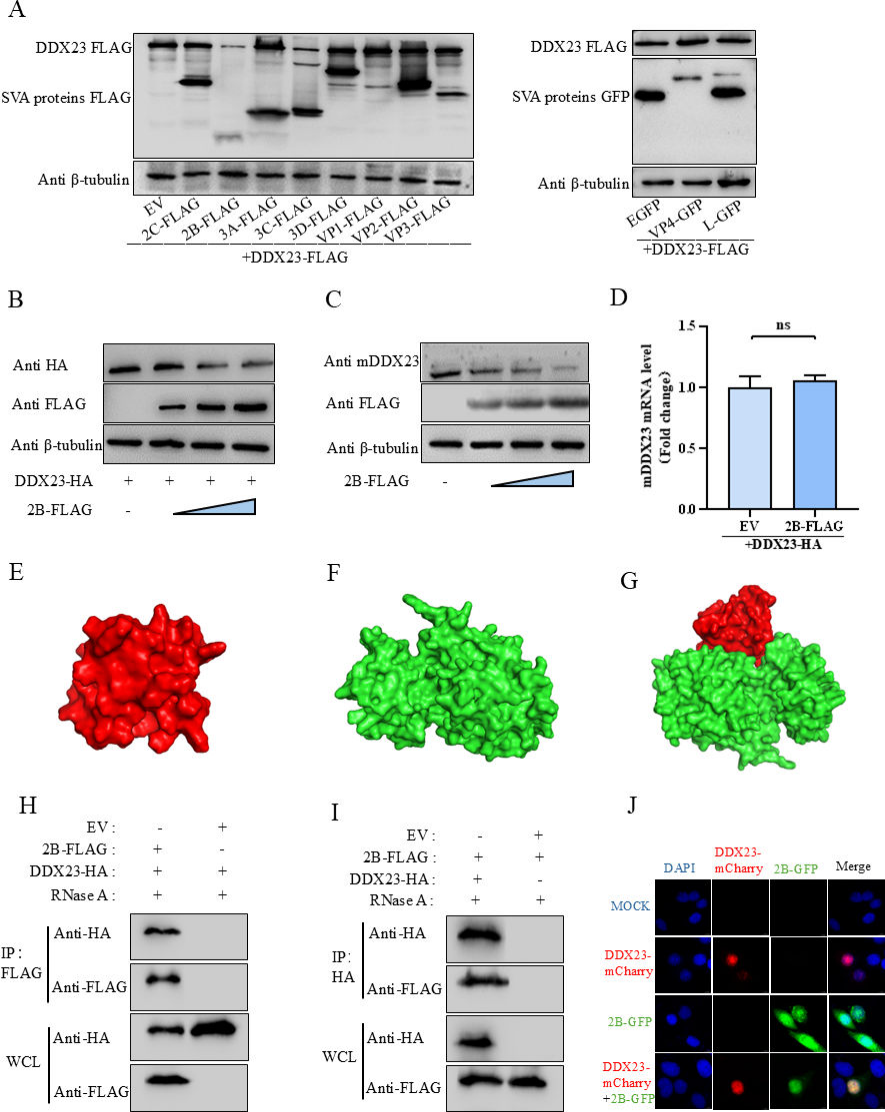
SVA-2B antagonizes DDX23 and interacts with DDX23. (**A**) DDX23 with FLAG label was co-transfected into HEK-293T cells with viral plasmid for western blot analysis. (**B**) DDX23 carrying HA label and SVA-2B plasmid with different doses of FLAG label were co-transfected into HEK-293T cells for western blot analysis. (**C**) Transfer SVA-2B plasmid to BHK-21 cells for western blot analysis to detect endogenous DDX23. (**D**) DDX23 and SVA-2B were co-transfected for qRT-PCR analysis (m means mouse). (**E**) 2B protein 3D model. (**F**) DDX23 3D model. (**G**) 3D results of predicted docking of DDX23 and SVA 2B proteins. (**H and I**) DDX23 with HA label and SVA-2B with FLAG label were transfected into HEK-293T cells for Co-IP test. (**J**) For the co-localization of DDX23 and SVA-2B, pmCharry-DDX23 (red light) and pEGFP-2B (green light) were co-transfected into BHK-21 cells and observed on the computer according to the IFA test procedure. All the above western blot experiments used β-tubulin as the upper sample control. All samples run in triplicate.

During the initial screening of the antiviral protein DDX23, we observed that endogenous mDDX23 underwent degradation following viral challenge, leading us to hypothesize that SVA exhibits protein antagonism against DDX23. To investigate this further, the plasmid pCA-DDX23-FLAG was co-transfected into HEK-293T cells along with individual SVA viral protein-expressing plasmids (L, VP1, VP2, VP3, VP4, 2B, 2C, 3A, 3B, 3C, and 3D) or an empty vector control. After 24 h, protein expression was analyzed via western blot. The results revealed that SVA 2B and 3C proteins inhibited DDX23 protein expression (Fig. 7A). Since there is much research on 3C mechanism, we will focus on 2B in the future. Subsequently, we transferred pCA-2B-Flag in a dose-dependent manner and detected the levels of endogenous and exogenous DDX23 protein in BHK-21 cells, which showed a dose-dependent decrease (Fig. 7B and C). Notably, SVA-2B did not affect the transcriptional level of DDX23 (Fig. 7D), suggesting that the degradation occurs post-transcriptionally. To further explore the interaction between SVA-2B and DDX23, computational modeling was employed to simulate their interaction (Fig. 7E through G). The results of docking using HDOCK showed that the docking score of model_1 with the lowest binding energy was −247.27, and the confidence score was 0.8749. The possibility of proteins binding in this form is high. The results show that there is a possibility of interaction between the two. To validate these findings experimentally, Co-IP assays were performed by co-transfecting HEK-293T cells with plasmids encoding pCA-DDX23-HA and pCA-2B-FLAG. The Co-IP results confirmed a physically indirect interaction between DDX23 and SVA-2B (Fig. 7H and I). Finally, to visualize the subcellular localization of these interactions, we constructed GFP-labeled 2B and mCherry-labeled DDX23 plasmids, transfected them into BHK-21 cells, and observed them with confocal laser microscopy. Confocal imaging results showed that DDX23 and SVA-2B were colocalized in the nucleus (Fig. 7J).

### SVA-2B degraded DDX23 by Caspase2 and Caspase3

**Fig 8:**
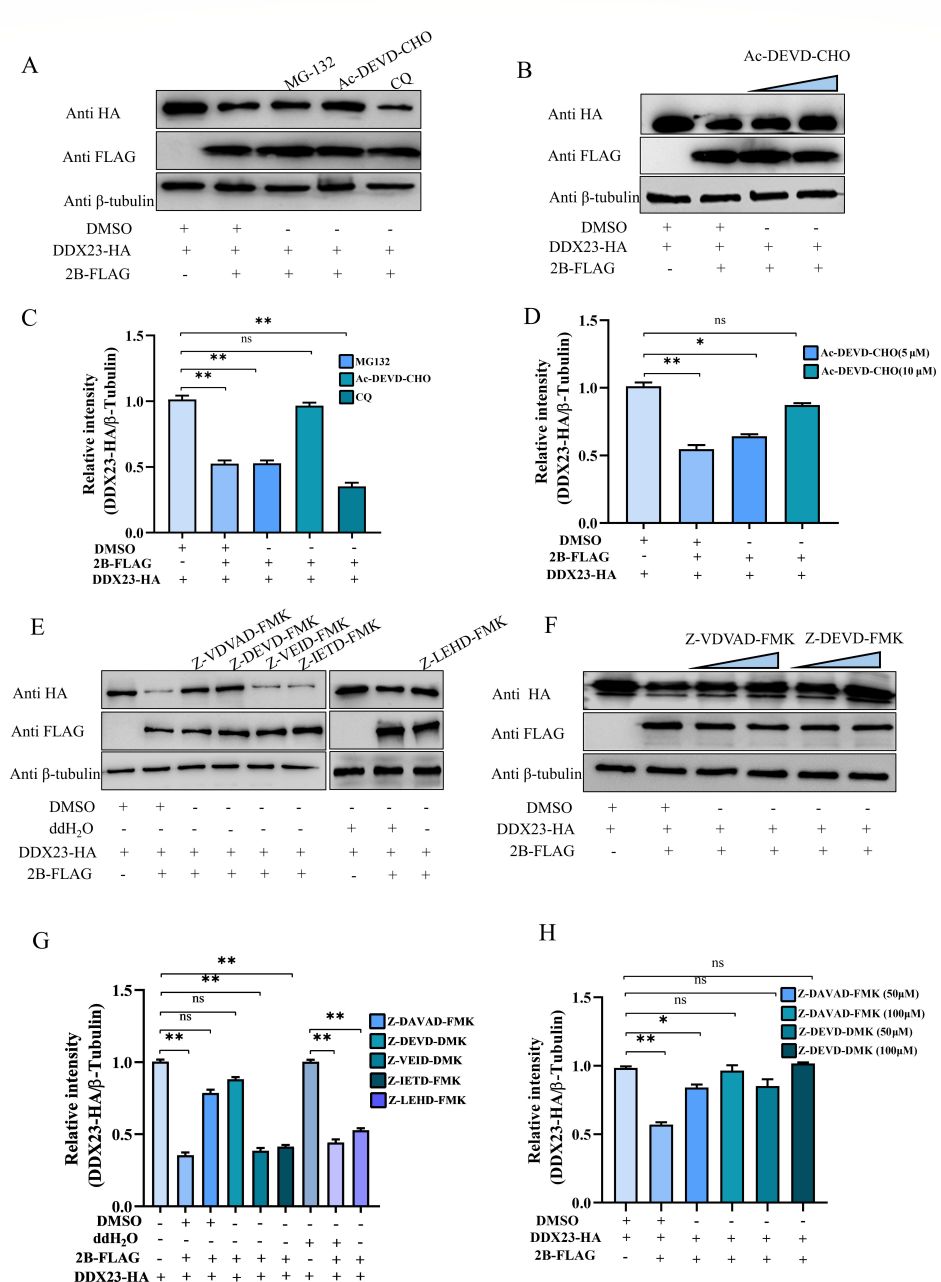
SVA-2B degraded DDX23 by Caspase2 and Caspase3. (**A and C**) pCA-2B-FLAG and pCA-DDX23-HA or EV were transfected into HEK-293T cells, and different inhibitors (MG132, CQ, or Ac-DEVD-CHO) were added 8 h later to affect the cells, while DMSO was set as a negative control group. Sixteen-hour cell samples were collected for western blot analysis to detect the changes in DDX23 protein. DDX23/β-tubulin protein levels are depicted as relative intensities. (**B and D**) Cells transfected with 2B and DDX23 were treated with different doses of Ac-DEVD-CHO, and cell samples were collected for western blot assay. DDX23/β-tubulin protein levels are depicted as relative intensities. (**E and G**) HEK-293T cells were transfected with pCA-2B-FLAG, pCA-DDX23-HA, or EV, and treated with different inhibitors (Z-VDVAD-FMK, Z-DEVD-FMK, Z-VEID-FMK, Z-IETD-FMK, and Z-LEHD-FMK) after 8 h, while DMSO and ddH_2_O were set as a negative control group. DDX23/β-tubulin protein levels are depicted as relative intensities. (**F and H**) Cells transfected with 2B and DDX23 were treated with different doses of Z-VDVAD-FMK and Z-DEVD-FMK, and cell samples were collected for western blot assay. DDX23/β-tubulin protein levels are depicted as relative intensities. All the above western blot experiments used β-tubulin as the upper sample control. All samples run in triplicate.

To determine the pathway by which SVA-2B degrades DDX23, plasmids pCA-DDX23-HA and pCA-2B-FLAG were co-transfected into BHK-21 cells. After transfection for 8 h, cells were treated with MG-132, Ac-DEVD-CHO, and CQ for 16 h, respectively. In these treatments, DDX23 protein levels recovered with interference from Ac-DEVD-CHO, while MG-132 and CQ failed to restore DDX23 protein levels (Fig. 8A through C). Meanwhile, the recovery effect of DDX23 protein was dose dependent with the dose of Ac-DEVD-CHO (Fig. 8B through D). In order to further clarify the mechanism, the Caspase degradation pathway was identified. Plasmids pCA-DDX23-HA and pCA-2B-FLAG were co-transfected into BHK-21 cells. After 8 h, cells were treated with specific inhibitors targeting Caspase-2/-3/-6/-8/-9 (Z-VDVAD-FMK, Z-DEVD-FMK, Z-VEID-FMK, Z-IETD-FMK, and Z-LEHD-FMK, respectively) for 16 h. Western blot analysis showed that DDX23 protein levels recovered under the interference of Z-VDVAD-FMK and Z-DEVD-FMK in these treatments, while other inhibitors failed to restore DDX23 protein levels (Fig. 8E through G). The recovery effect of DDX23 protein was dose dependent with the doses of Z-VDVAD-FMK and Z-DEVD-FMK (Fig. 8F through H). These findings suggest that Caspase-2 and Caspase-3 play the key role in SVA-2B-induced degradation of DDX23.

### The 44th and 45th amino acid residues of 2B are the determinants for DDX23 degradation

**Fig 9:**
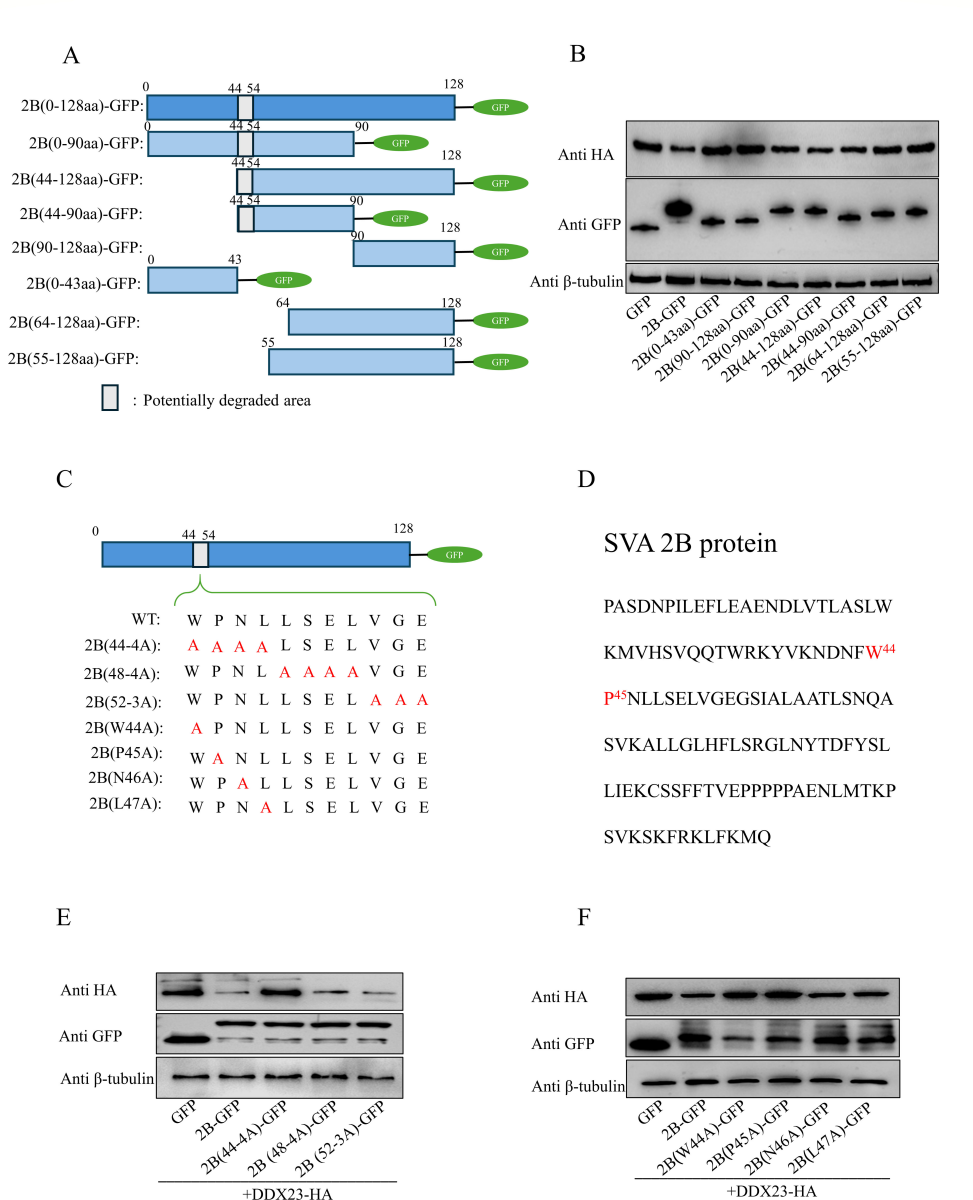
The 44th and 45th amino acid residues of 2B are the key sites for inducing degradation. (**A**) Construct a schematic diagram of SVA-2B truncation. (**B**) HEK-293T cells were transfected with SVA-2B truncated body and plasmid pCA-DDX23-HA, and samples were collected 24 h later for western blot analysis. (**C**) Construct a schematic diagram of SVA-2B mutant. (**D**) Schematic diagram of amino acids at SVA-2B action sites. (**E and F**) SVA-2B mutant and plasmid pCA-DDX23-HA were co-transfected into HEK-293T cells, and samples were collected 24 h later for western blot analysis. All the above western blot experiments used β-tubulin as the upper sample control. All samples run in triplicate.

In order to further explore the region where 2B exerts the degradation function, we constructed a series of 2B truncations with GFP flags. The scopes and names of these truncates are 0–90 aa, 44–128 aa, 44–90 aa, 90–128 aa, 0–43 aa, 55–128 aa, and 64–128 aa (Fig. 9A). The plasmid was co-transfected into HEK-293T cells with an HA-labeled DDX23 expression plasmid. After 24 hours, the cell lysate was collected for western blot analysis. The results show that the 44–54 amino acid regions of 2B are potential functional regions (Fig. 9B). Subsequently, we performed multi-site and single-site mutations targeting this region to further identify the site of action. The mutants were named 2B(44-4A), 2B(48-4A), 2B(52-3A), 2B(W44A), 2B(P45A), 2B(N46A), and 2B(L47A). The schematic diagram is shown in Fig. 9C. Through co-transfection experiments, we identified the 44 and 45 amino acid residues of 2B as key sites for inducing degradation of DDX23 (Fig. 9D through F).

### Construction and identification of recombinant viruses mutated at functional sites of 2B and 3A proteins

**Fig 10:**
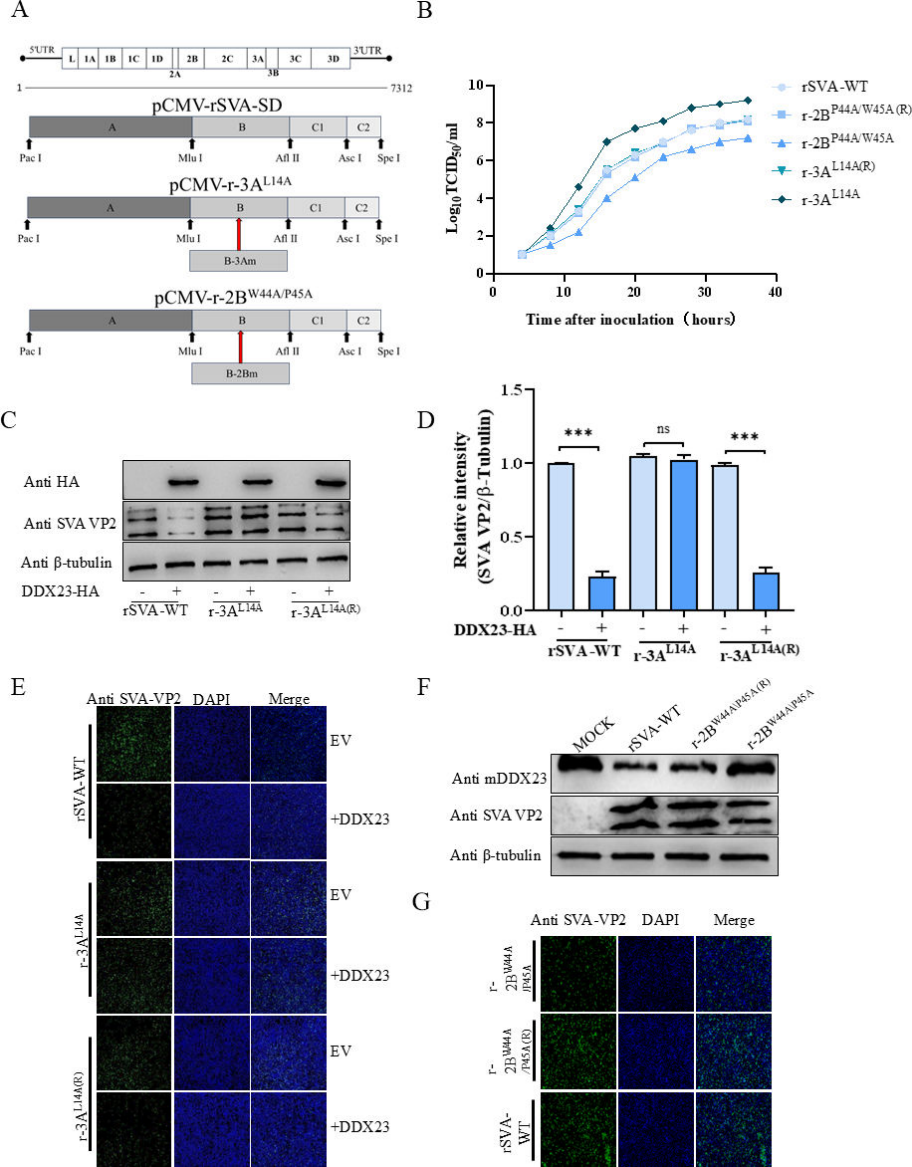
Construction and identification of recombinant viruses mutated at functional sites of 2B and 3A proteins. (**A**) Schematic construction of infectious clonal plasmids 2B and 3A. (**B**) Growth curve determination of rSVA-WT, r-3A^L14A^, r-3A^L14A (R)^, r-2B^W44A/P45A^, and r-2B^W44A/P45A(R)^. (**C and D**) BHK-21 cells transfected with pCA-DDX23-FLAG or EV were infected with rSVA-WT, r-3A^L14A^, and r-3A^L14A (R)^, respectively; 18 h later, cell lysate was collected 24 h later for western blot analysis. VP2 and DDX23 were detected with anti-VP2 and anti-FLAG antibodies. VP2/β-tubulin protein levels are depicted as relative intensities. (**E**) IFA results after rSVA-WT, r-3A^L14A^, and r-3A^L14A(R)^ infection. (**F**) The untreated BHK-21 cells were infected with rSVA-WT, r-2B^W44A/P45A^, r-2B^W44A/P45A(R)^, and cell lysate was collected for western blot analysis 24 hours later. Anti-VP2 and anti-DDX23 antibodies were used to detect VP2 and endogenous DDX23. (**G**) IFA results after rSVA-WT, r-2B^W44A/P45A^, and r-2B^W44A/P45A(R)^ infection. All the above western blot experiments used β-tubulin as the upper sample control. All samples run in triplicate. ***, *P* < 0.001; ns, *P* > 0.05.

In order to evaluate the effects of SVA-2B and SVA-3A gene functional sites on viruses, viral plasmids containing 2B and 3A mutations were constructed using the infectious clonal plasmids of SVA respectively, as shown in Fig. 10A. After transfection into BHK-21 cells, the virus was successfully rescued after cell passage and named r-2B^W44A/P45A^ and r-3A^L14A^, respectively. Two revertants, r-2BW44A/P45A(R) and r-3AL14A(R), were rescued from the corresponding r-2BW44A/P45A and r-3AL14A infectious cDNA clones. Growth curve analysis showed that r-2B^W44A/P45A^ replicated more slowly than r-2B^W44A/P45A(R)^ and rSVA-WT, while r-3A^L14A^ replicated more quickly than r-3A^L14A(R)^ and rSVA-WT (Fig. 10B). BHK-21 cells overexpressing DDX23-FLAG plasmid were then inoculated with 0.05 MOI virus rSVA-WT, r-3A^L14A^, and r-3A^L14A(R)^, respectively, and protein samples were collected 16 h later for western blot detection. The results showed that viral protein expression in the r-3A^L14A^ group was significantly recovered compared with the rSVA-WT group and the r-3A^L14A(R)^ group (Fig. 10C). Gray value scanning further supports the experimental results (Fig. 10D). BHK-21 cells of the same treatment were detected by indirect immunofluorescence (Fig. 10E), and the results were consistent with western blot. BHK-21 cells were then inoculated with 0.05 MOI rSVA-WT, r-2B^W44A/P45A^, and r-2B^W44A/P45A(R)^, respectively, and cell samples were collected 16 h later for western blot analysis. Results showed partial recovery of endogenous DDX23 in the r-2B^W44A/P45A^ group inoculated with recombinant virus compared to the rSVA-WT and r-2B^W44A/P45A(R)^ groups (Fig. 10F). Unfortunately, the endogenous DDX23 antibody prepared by us could not be used for indirect immunofluorescence detection. We could only observe that after inoculation with r-2B^W44A/P45A^, the number of cellular viruses decreased significantly compared with the other two groups. This suggests that r-2B^W44A/P45A^ reduces the degradation of DDX23, causing DDX23 to continue to play a role in inhibiting the virus, resulting in an increased inhibitory effect of DDX23 on the virus and a decrease in viral infectivity (Fig. 10J). The results indicate that mutating L14 in the 3A protein of the SVA virus significantly enhances viral replication, whereas mutating W44 and P45 in the 2B protein inhibits it.

## DISCUSSION

SVA, initially considered non-pathogenic and non-infectious to animals when it was isolated, has now transformed to infect pig herds and spread widely, which has affected the green and healthy development of the global farming industry. The clinical manifestations of SVA closely resemble those caused by FMDV and VSV, including vesicular disease, neonatal piglet mortality, and persistent infections, all of which pose substantial challenges to the pig farming industry (32, 33). Because no effective antiviral drugs or commercially available vaccines have been developed to combat SVA, the prevalence of SVA and its impact on the breeding industry are increasing. Therefore, our research is of great significance for the green and healthy development of the pig industry. In this study, we provide the first evidence of the inhibitory effect of the host gene DDX23 on SVA, identifying the associated proteins, pathways, and degradation sites involved. Furthermore, we elucidate the mechanisms by which SVA evades the antiviral activity of the host gene DDX23, offering novel insights into the virus-host interaction.

The DEAD-box helicase family, a significant branch of the helicase superfamily, is ubiquitous in biology and plays a crucial role in various life activities. Numerous studies have demonstrated the involvement of DEAD-box helicase family proteins in viral infections, highlighting their importance in antiviral defense mechanisms. For instance, overexpression of DDX4, a member of this family, has been shown to enhance type I IFN-mediated signaling pathways, thereby inhibiting the protein expression of several viruses, including VSV and herpes simplex virus (34). Similarly, DDX6 has been reported to assist RIG-I in sensing enterovirus 71 (EV71) invasion, enhancing IFN-I expression, and suppressing viral proliferation (35). Another member, DDX39A, contributes to antiviral defense by binding to conserved regions in the chikungunya virus genome, thereby affecting translation and controlling infection. Among these helicases, DDX23 plays particularly diverse roles in RNA transcription, translation, splicing, RNA-binding regulation, and modulation of viral replication (18–20). Recent studies have identified DDX23 as a conserved double-stranded RNA sensor that forms a complex with MAVS or Toll/interleukin-1 receptor domain-containing adaptor protein (TRIF). This complex activates downstream innate immune signaling pathways, thereby inhibiting VSV infection (25). These findings collectively suggest that DDX23 exerts antiviral effects through multiple mechanisms. In the present study, we investigated the role of DDX23 in SVA infection. Together, these findings suggest that DDX23 exerts its antiviral effects through multiple mechanisms. It is well known that most RNA uncoupling enzyme antiviral mechanisms rely on interaction with viral RNA. For example, FMDV, a member of the “family of small RNA viruses,” has been shown to interact with the internal ribosome entry site (IRES) structural domain III of its genome during infection, inhibiting FMDV replication by downregulating IRES-mediated genome translation (26). Although uncoupling enzymes that affect protein stability are rare, recent reports have shown that the uncoupler enzyme DDX10 is able to interact with the circovirus type 3 Cap protein and thereby inhibit viral replication (36). Consistent with this, the present study demonstrated that DDX23 reduced viral proliferation by targeting and degrading the SVA-3A protein. Nevertheless, since DDX23 has been shown to activate innate immunity, it is still necessary to confirm whether innate immunity is involved in the process of DDX23 anti-SVA in BHK-21 cells. Reports have shown that cells routinely tested for innate immunity, such as PK-15 cells, can have up to 100 or even 1,000 times higher transcript levels after IFN or ISG activation compared to before activation (37, 38). However, in the present study, we further overexpressed DDX23 in BHK-21 cells and detected only 2.75- and 3.3-fold increases in mRNA levels of IFN-β and ISG15 after SVA infection (Fig. S2). Therefore, we hypothesized that the inhibition of SVA replication by DDX23 in BHK-21 cells does not depend on its function of activating innate immunity, and this antiviral effect is more dependent on interactions with and targeted degradation of 3A. Overexpression of DDX23 significantly inhibited SVA proliferation in BHK-21 cells, whereas deletion of DDX23 enhanced viral proliferation. Viral infection typically involves sequential steps, including binding, entry, replication, and release (39). Our results suggest that DDX23 does not affect the binding or entry phase of the SVA infection cycle but inhibits the viral replication phase. Further analysis showed that DDX23 inhibited SVA replication by degrading viral SVA-3A protein. When we investigated the antagonistic effect of SVA on DDX23, we found that the level of DDX23 protein was significantly decreased in BHK-21 cells infected with SVA, while the level of DDX23 transcription was significantly increased. We determined that the cells were trying to increase transcription levels to counteract the degradation of DDX23. Consistent with this, our follow-up study confirmed that the reduction of DDX23 protein is due to the targeted degradation of DDX23 protein by the 2B protein expressed during SVA proliferation. It can be foreseen that the virus allows the host to respond to the virus (including DDX23 transcription) in the early stages of infection, avoiding premature triggering of cell apoptosis or strong inflammatory reactions and buying time for self-replication. This is also one of the reasons for the increase in DDX23 transcription levels after SVA infection. Subsequently, when the virus enters the late stage of replication, DDX23 is degraded by the 2B protein to achieve antagonistic escape. However, our measurement results indicate that 2B does not affect the mRNA level of DDX23 and only degrades DDX23 through the apoptotic pathway at the protein level. Consistent with this, HCV NS3/4A protease can specifically recognize specific cleavage sites on the MAVS protein and directly cleave the MAVS protein itself. This cleavage destroys the key functional domains of MAVS, and the cleaved MAVS fragments lose their signaling function and are typically rapidly degraded and cleared by the cell’s proteasome system. This process does not involve inhibition of MAVS gene transcription, nor does it involve disruption of MAVS mRNA stability (40). Therefore, this degradation strategy precisely reflects the “survival wisdom” evolved by viruses over billions of years, achieving maximum destruction through minimal disturbance. It does not affect host transcriptional regulation to avoid triggering secondary alarm systems and utilizes host degradation machines to achieve precise strikes. During the confrontation between SVA and DDX23, we revealed that DDX23 physically and indirectly interacts with SVA-2B and SVA-3A, respectively, through electronic simulation, Co-IP, and laser confocal experiments, and DDX23 is co-located with 2B and 3A in the nucleus. These findings provide insights into the antiviral effects of DDX23 on SVA and suggest that SVA evolved to evade the antiviral function of DDX23.

Eukaryotic cells utilize three primary protein degradation pathways: the lysosomal pathway, the ubiquitin-proteasome pathway, and the caspase pathway. The ubiquitin-proteasome pathway has been implicated in the regulation of viral replication, as evidenced by the role of the host protein ubiquitin-conjugating enzyme UBE2L6 in positively regulating SVA replication through this pathway (41). Similarly, the lysosomal pathway has been shown to mediate protein degradation, as demonstrated by the interaction between the hnRNP K protein and PEDV-N protein, which induces the degradation of the latter via the autophagic lysosomal pathway (42). The caspase pathway, on the other hand, is regulated by the Caspase family of proteins, which are cysteine proteases existing as zymogens composed of four heterotetrameric subunits of varying sizes (43–45). These enzymes are not only central to apoptosis but also cleave various intracellular proteins during cellular processes. For instance, the non-structural protein 2B of SVA-related enterovirus EV-71 has been reported to inhibit KPNA1 degradation in cells pretreated with Caspase-3 inhibitors (46). Similarly, Caspase-3 inhibitors were found to suppress the degradation of MAVS induced by SVA-2B. Although the amino acid sequences of EV-A71-2B and SVA-2B are not highly similar (data not shown), their secondary structures exhibit notable resemblance, suggesting a potential functional relationship between the 2B protein of small RNA viruses and Caspase family proteins.

In order to explore these pathways of degradation, we mediated three inhibitors of protein degradation pathway MG132 (ubiquitin-proteasome pathway), Ac-DEVD-CHO (caspase pathway), and CQ (lysosomal pathway) into DDX23 and SVA-2B/3A for co-transfection assay. Interestingly, the use of Ac-DEVD-CHO partially reversed the degradation of SVA-3A by DDX23 and DDX23 by SVA-2B. This observation indicates that the degradation of SVA-3A by DDX23 and DDX23 by SVA-2B is mediated, at least in part, through the caspase pathway. To identify the specific Caspase proteins involved in these processes, we treated cells with inhibitors targeting Caspase-2/-3/-6/-8/-9. The results demonstrated that Caspase-2 and Caspase-3 were responsible for mediating the degradation of DDX23 by SVA-2B, while Caspase-3 and Caspase-6 mediated the degradation of SVA-3A by DDX23. These findings underscore the pivotal role of specific Caspase family proteins in regulating the degradation of both host and viral proteins, thereby advancing our understanding of the molecular mechanisms governing SVA-host interactions. Regretfully, we were not able to overexpress or knock out these nodal proteins to further support our results, but molecule treatments support the role of Caspases in DDX23 viral protein interactions. Although this paper confirms the existence of a relationship between DDX23 and 3A degradation and cysteine asparaginase, it is not yet clear how their cysteine asparaginase is involved in their degradation, and we speculate that they achieve this by interacting with cysteine asparaginase and triggering apoptosis. In conclusion, these interactions may be indirect, and further studies are needed to better understand the interrelationships between DDX23, viral proteins, and cysteinyl asparaginase.

The primary amino acid sequence of the 2B protein exhibits significant divergence from that of viruses within the small RNA family; however, its secondary structure consistently contains a helix-helix-helix domain (47). Notably, studies on Cucumber Mosaic Virus have demonstrated that the 2B protein functions as an RNA-silencing suppressor, thereby establishing its role as a determinant of cytomegalovirus (CMV) pathogenicity (48). Similarly, the 2B protein of FMDV has been shown to facilitate immune evasion by recruiting E3 ubiquitin ligase 115 (RNF125) and promoting the degradation of RIG-I via the ubiquitin-proteasome pathway. This immune suppression mechanism is critically mediated by the 126-154 amino acid region of FMDV-2B (49). In contrast, studies on the 3A protein have revealed its high degree of conservation across all picornaviruses due to its direct homology (50). The 3A protein of SVA, in particular, possesses a transmembrane helical structure characterized by a highly hydrophobic region, which is hypothesized to enable its function as a membrane-binding protein with regulatory effects on host cell membranes (51). Furthermore, it has been reported that the 3A protein can independently induce mitochondrial autophagy, underscoring its multifaceted role in viral pathogenesis (52). There are also related reports on the influence of special sites of viral proteins on viruses. For example, K169aa and K321aa of SVA-3D are ubiquitization sites mediated by anti-SVA gene UBE2L6, and the virus significantly proliferates after mutation of their sites (41). The 2C protein of SVA is anchored to mitochondria through direct interaction with mitochondrial TUFM protein, and TUFM is subjected to K27-linked ubiquitin modification catalyzed by E3 ubiquitin ligase RNF185. The regions of amino acids 231–321 aa in 2C protein and 56–252 aa in TUFM protein are the key regions for their interaction (51). These results fully demonstrate that key regions and sites of viral proteins play an indispensable role in the process of viral proliferation. Therefore, in our subsequent study, we used truncation and mutation techniques to identify tryptophan and proline as functional sites for degrading DDX23 at positions 44 and 45 of SVA-2B. At the same time, DDX23 targets the 14-position leucine of SVA-3A, causing its degradation. In the process by which DDX23 and 3A are degraded, we found that the degradation is lost after mutating 3A and 2B. However, this bi-directional process involves the degradation of both proteins, and it is not known whether this degradation process is dependent on the interaction between the two. We therefore visualized the interaction between DDX23 and the 3A and 2B mutants using the HDOC server (Fig. S3). We found that the 3A and 2B mutants are still able to interact with DDX23, but the key sites described above are not located in the region of interaction, so they do not escape degradation by avoiding interaction with DDX23. The region of interaction is independent of the functional region that exerts degradation. How these mutants avoid degradation needs to be further explored, but it is not the focus of this study. Based on these findings, we constructed and rescued two recombinant viruses: the 2B double-site mutant virus (r-2B^W44A/P45A^) and the single-point mutant virus (r-3A^L14A^) of 3A. Experimental results revealed that the replication capacity of the 2B recombinant virus was significantly weakened, accompanied by a notable reduction in its inhibitory effect on DDX23. Consequently, the antiviral effect of this recombinant virus against DDX23 at the cellular level was markedly diminished. In contrast, the replication ability of the 3A recombinant virus was significantly enhanced, and it was observed to evade the inhibitory effect of DDX23 in repeated experimental tests. However, this study did not extend to animal models to further investigate the pathogenicity changes of the mutant strains. Despite this limitation, the findings provide a valuable foundation for future research, particularly in the development of attenuated live vaccine targets for SVA.

**Fig 11:**
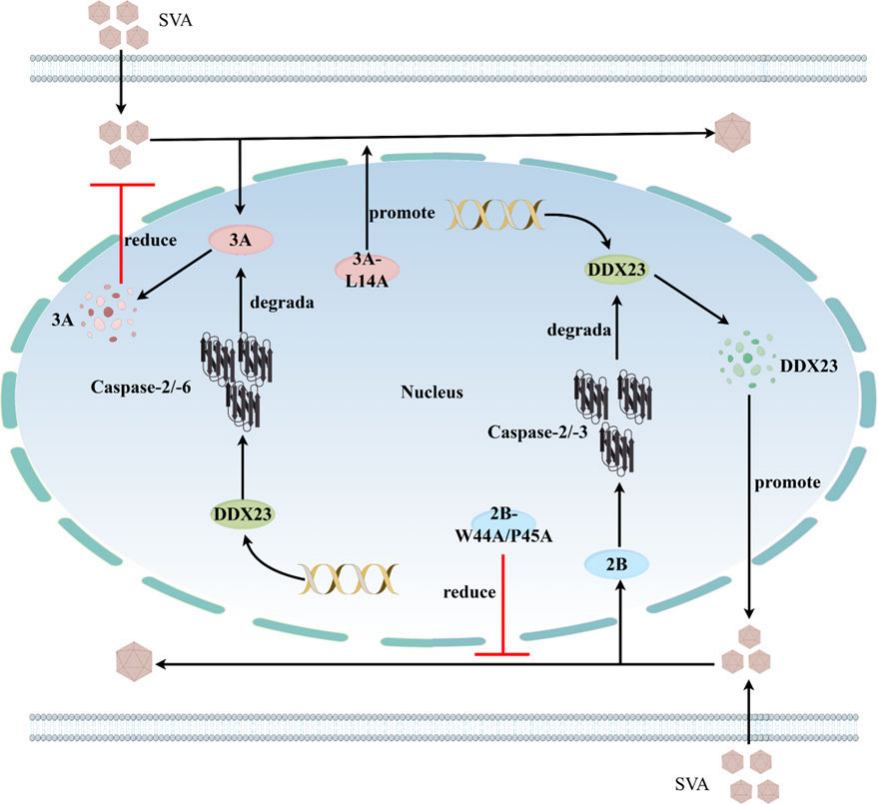
After SVA enters the cell, the host antiviral gene DDX23 activates Caspase-2/-6 to attack leucine at the 14th position of SVA-3A, thereby inhibiting viral prolifx`eration. At the same time, the virus turned on the self-protection mechanism, and the viral non-structural protein 2B also degraded DDX23 through the Caspase-2/-3 pathway, in which tryptophan 44 and proline 45 of 2B were the key sites for the degradation of DDX23.

In conclusion, this study demonstrated that DDX23 plays a critical role in degrading the SVA-3A protein, thereby influencing the stability of SVA. Mechanistically, DDX23 targets leucine 14 of SVA non-structural protein 3A, promoting its degradation through Caspase-3 and Caspase-6 in the Caspase pathway. To counteract this antiviral factor, SVA employs its non-structural protein 2B to suppress DDX23 expression, thereby promoting viral replication. Further mechanistic analysis revealed that SVA-2B mediates the degradation of DDX23 by utilizing tryptophan and proline residues at positions 44 and 45, respectively, through the activation of Caspase-2 and Caspase-3 in the Caspase pathway (Fig. 11). These findings provide valuable insights into the molecular interplay between SVA and host antiviral defenses, highlighting the dual roles of DDX23 and SVA in regulating viral replication and host immune evasion.

## MATERIALS AND METHODS

### Cells, viruses, and reagents

PK-15, HEK-293T cells, and BHK-21 cells were cultured in Dulbecco’s modified Eagle’s medium (DMEM, Gibco, USA) supplemented with 10% fetal bovine serum (Hyclone) and 100 µg/mL penicillin and streptomycin. All the cells were cultured at 37°C in a humidified 5% CO_2_ incubator. The SVA strain SVV-CH-SD (GenBank accession no. MH779611) was passaged in BHK-21 cells and maintained in our laboratory. Anti-HA and Anti-GFP antibodies were purchased from Proteintech Biotechnology, USA, and Anti-FLAG and anti-β-tubulin were purchased from Abmart, China. The polyclonal antibodies for SVA VP2 and DDX23 were made in our laboratory. Horseradish peroxidase (HRP)-labeled goat anti-mouse and goat anti-rabbit antibodies were purchased from Beyotime, China. MG132 (53) (Selleck, S2619), Ac-DEVD-CHO (54) (Selleck, S7901), Chloroquine (55) (Selleck, S6999), Z-VDVAD-FMK (56) (APExBIO, A1922), Z-DEVD-FMK (54) (Selleck, S7312), Z-VEID-FMK (57) (APExBIO, A1923), Z-IETD-FMK (58) (Selleck, S7314), and Z-LEHD-FMK (58) (Selleck, E7038).

### Plasmids

Using the gene primers for DDX3X/5/6/19A/21/23/27/46/56 listed in Table S1, cDNA from PK-15 cells was amplified and cloned into modified pCAGGS label containing either 3 'FLAG or HA. DDX23 was also inserted into pmCherry-N1 vector (colocalization experiment). SVA protein L, VP1, VP2, VP3, VP4, 2B, 2C, 3A, 3C, 3D, and SVA-infected clone plasmid pCMV-rSVA were donated by Animal Bacteriology Laboratory of Nanjing Agricultural University. Primers in Table S1 were used to amplify the 3A and 2B truncated segments using the viral plasmid cDNA as a template. The 3A amplification product was cloned into the plasmid pCAGGS inserted with the 3 'FLAG marker, and the 2B amplification product was cloned into the pEGFP-N1 vector (the 2B protein was too small). The 3A and 2B mutants were constructed by overlap PCR, whereas the 3A(10-5A), 3A(L14A), 3A(A13G), and 3A(E12A) mutants were constructed by normal PCR amplification.

### Construction of a DDX23 knockout cell line

Three sgRNA sequences were designed using the online CRISPR design tool (https://www.benchling.com) to target the exons of DDX23. A Bbs I restriction site was added at the sgRNA 5' terminus during synthesis. Double-stranded nucleotide fragments were obtained by annealing the synthesized sgRNA oligonucleotides at 95°C for 5 minutes, and then the temperature was ramped from 95°C to 25°C at a rate of 0.1°C/S. The annealed fragments were digested with Bbs I and cloned into the CRISPR-Cas9 vector pX-459 to generate pX-459-DDX23-sgRNA1, pX-459-DDX23-sgRNA2, and pX-459-DDX23-sgRNA3.

BHK-21 cells at 50% confluence in 12-well plates were transfected with empty vector control or recombinant plasmid using Lipofectamine 3000 transfection reagent according to the manufacturer’s instructions. After 48 h, the transfected cells were treated with 0.6 mg/mL puromycin, and the cells were passaged every 48 h with the administration of puromycin. In order to isolate monoclonal cells, the cells under the action of puromycin were limitedly diluted to 10–100 cells/mL and planted in 96-well cell plates. After incubation for about 1 week, colonies formed from single cells could be identified and were harvested for genomic analysis. The genomic DNA was extracted from the knockout cells with the Universal MiniBEST Genomic DNA Extraction Kit (TaKaRa). The genomic DNA of the cells cultured from a single clone was amplified with the primers based on the genomic DNA sequence of DDX23 (Table S2). Ten clone-derived samples were sequenced to ensure that the deletion was established in the cell line. The western blot analysis was performed to confirm that DDX23 was not expressed in the DDX23-knockout cell line. WT BHK-21 cells were used as the control.

### Virus titration

BHK-21 cells were seeded in 96-well plates and then infected with 10-fold serial dilutions of SVA virus, with a total of eight replicates. After 1 h at 37°C, the cells were washed with PBS, and complete medium was added. The plates were incubated for 48–72 h in a cell culture incubator. SVA titers were calculated using the Reed-Muench method.

### RNA extraction and real-time fluorescence quantitative PCR

Total RNA was extracted from cells using the E.Z.N.A. Total RNA Kit (Omega Bio-Tek, Inc., Norcross, GA, USA) and then reverse transcribed using the HiScript II First Strand cDNA Synthesis Kit (Vazyme Biotechnology Co., Ltd., Nanjing, China). qRT-PCR was performed using the SuperReal PreMix Plus (SYBR Green; TIANGEN BIOTECH Co., Ltd.) according to the manufacturer’s instructions. β-tubulin was used as the reference gene, and all data are expressed as relative fold change (calculated using the 2^−ΔΔCT^ method). All primers for qRT-PCR are presented in Table S3.

### Western blot assay

Radioimmunoprecipitation assay (RIPA) (Beyotime, China) and phenylmethanesulfonyl fluoride (PMSF) (biosharp, China) were mixed thoroughly in a 100:1 ratio for the preparation of the lysate and placed on ice for later use. BHK-21 or HEK-293T cells were washed with PBS and lysed on ice with lysis solution for 15 min. Protein samples containing equal amounts were separated by 10% SDS-PAGE and transferred to polyvinylidene fluoride (PVDF) membranes. After transfer, PVDF membranes were closed with 5% skim milk powder (diluted with phosphate buffered saline Tween 20 [PBST]) for 1 h at room temperature, washed three times with PBST, and then incubated with the following primary antibodies: anti-SVA VP2-protein (1:3,000), anti-β-tubulin (1:5,000; sc-47778, Santa Cruz, USA), anti-FLAG (1:5,000, Abmart), anti-HA (1:5,000, Abmart), and anti-DDX23 (1:10,000, Abmart). The membranes were incubated at room temperature for 2 h or overnight at 4°C. The membranes were then washed three times with PBST and then incubated with HRP-conjugated goat anti-mouse and anti-rabbit IgG (H + L) secondary antibodies for 1 h at room temperature (1:5,000; Beyotime, China). Binding proteins were exposed with the ECL kit (Tanon, China).

### 3D modeling and protein-protein interactions

The three-dimensional structures of DDX23 protein and SVA-3A/−2B were constructed by AlphaFold, and protein-protein molecular docking was performed using the ClusPro 2.0 protein docking web server (https://cluspro.bu.edu/login.php). The protein three-dimensional structure and docking results were visualized by PyMol.

### Co-immunoprecipitation

HEK-293T cells were lysed in RIPA buffer containing PMSF. The lysate was centrifuged at 8,000 rpm for 5 min, and the supernatant was incubated with 20 µL protein A/G agarose beads (Beyotime, China) for 1 h at 4°C. Centrifuge the lysate at 2500 rpm for 5 min. Collect the supernatant and incubate with 2 µg of the appropriate mouse antibody and 40 µL of protein A/G agarose beads overnight at 4°C on a roller. Sepharose beads were collected by centrifugation, washed five times with 1 mL of lysis buffer, and then suspended in 100 µL of lysis buffer. Finally, whole cell lysates and immunoprecipitates are used for western blot.

### Cell viability assay

Cell viability was measured using the Cell Counting Kit-8 (Beyotime) following the manufacturer’s instructions. Results are expressed relative to control cells, which were defined as 100% viable.

### Inhibitor treatment

BHK-21 cells were cultured in 12-well cell culture plates until 70% confluent and transfected with related plasmids for 8 h. Then MG-132, CQ, Ac-DEVD-CHO, Z-VDVAD-FMK, Z-DEVD-FMK, Z-VEID-FMK, Z-IETD-FMK, Z-LEHD-FMK, DMSO, or ddH_2_O were treated and cultured for 16 h, and the cell proteins were collected for western blot.

### Construction of a viral infectious clone plasmid

The construction strategy is shown in Fig. 1. Using pCA-2B-FLAG as the template, the 2B mutant fragment was amplified by using the primers in Table S1 through Overlap technology. Since this fragment was located in the B fragment of pCMV-rSVA-SD, the product was named B-2BM. pCMV-rSVA-SD was cut with AflII and MluI enzymes. Overlap PCR product was connected to the cut pCMV-rSVA-SD, named pCMV-r-2B^P44A/W45^, and then the recombinant virus recovery plasmid was constructed by the same method and named pCMV-r-2B^W44A/P45A(R)^. The 3A infectious clone plasmid was constructed by the same method.

### Save the mutant viruses

BHK-21 cells were digested by pancreatic enzymes and spread on 12-well cell culture plates. When the cell fusion degree reached 80%, plasmids pCMV-r-2B^W44A/P45A^ and plasmids pCMV-r-2B^W44A/P45A(R)^ were transfected into BHK-21 cells according to the procedure described by Lipofectamine 3000 transfection reagent. Meanwhile, an empty plasmid transfection control group was set up. The cells were cultured at 37°C and 5% CO_2_ and observed day by day. When 80% cytopathic effect (CPE) was present in the test group, the virus solution was repeatedly frozen and thawed three times, and the virus was harvested at 12,830 × *g*, centrifuged, divided into supernatant, frozen, and stored at −80°C for later use, and named r-2B^W44A/P45A^ and r-2B^W44A/P45A(R)^. 3A mutant infectious clone virus rescue ibid.

### Viral growth curves

BHK-21 cells were seeded on 24-well cell culture plates until they grew into monolayers and inoculated with rSVA-WT, r-2B^W44A/P45A^, and r-2B^W44A/P45A(R)^ at 100 µL per well. After treatment at 37°C for 1 h, the venom was sucked out, and 500 µL 2% fresh DMEM solution was added for further culture. The cell culture supernatant was collected at 4, 8, 12, 16, 20, 24, 28, 32, and 36 h after infection and stored at −80°C. After all the time points were collected, TCID_50_ was measured, and the growth curve was drawn. 3A recombinant virus was determined by the same method.

## Data Availability

All data generated or analyzed during this study are included in this article and its supplemental material. All other original data supporting the conclusions of this study can be obtained from the corresponding authors upon reasonable request.

## SUPPLEMENTAL MATERIAL

Supplemental figures jvi.00761-25-s0001.pdf Figures S1 to S3.

Table S1 jvi.00761-25-s0002.xlsx Primers for generating DDX family protein expression plasmid, 3A luminescent plasmid, 2B luminescent plasmid, 3A truncated body, 2B truncated body, 3A mutant, 2B mutant, r-2BW44A/P45A, and r-3AL14A.

Table S2 jvi.00761-25-s0003.xlsx Primers for generation of DDX23-KO cells.

Table S3 jvi.00761-25-s0004.xlsx Primers used for RT-qPCR.

ASM does not own the copyrights to Supplemental Material that may be linked to, or accessed through, an article. The authors have granted ASM a non-exclusive, world-wide license to publish the Supplemental Material files. Please contact the corresponding author directly for reuse.
