# Supplementary material for: 3A and 2B proteins of SVA play chess game with host restriction factor DDX23 by apoptotic pathway
Source: J Virol. 2025 Sep 16;99(10):e00761-25. doi: 10.1128/jvi.00761-25 (PMC12548459; doi:10.1128/jvi.00761-25)
Supplement: Supplemental figures — Figures S1 to S3. [file jvi.00761-25-s0001.pdf]

Supplemental Figure1

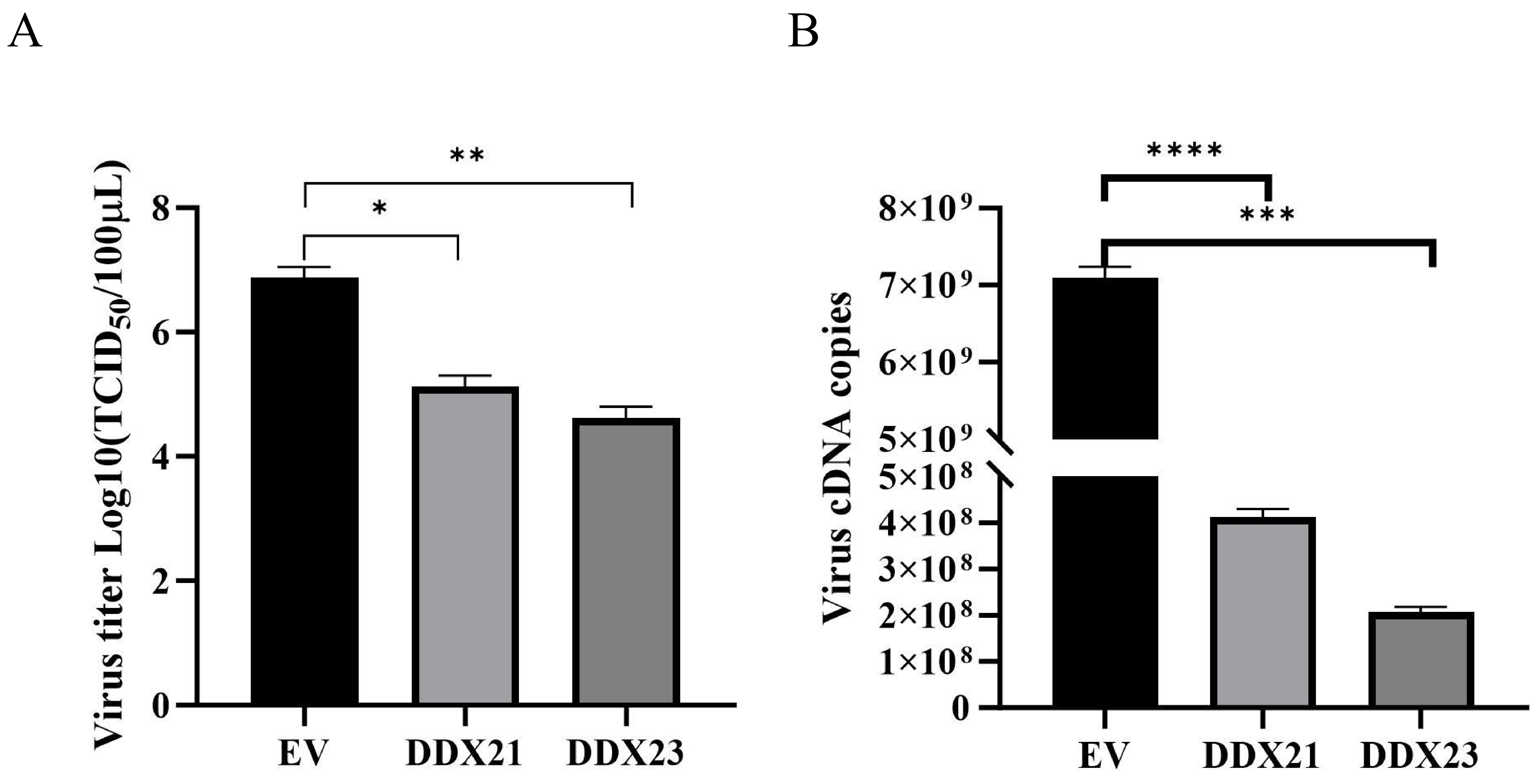

Supplemental Figure1. Determine the effect of DDX21 and DDX23 on SVA replication by detecting virus copy number and titer. (A)BHK-21 cells transfected with pCA-DDX21-FLAG, pCA-DDX23-FLAG or empty vector were infected with SVA 24 hours later, and the cell supernatant was collected for virus titer analysis 16 hours later; (B) Dilute the pCA-VP1 plasmid ten times continuously to generate a standard curve. Determine the cDNA copies of samples pCA-DDX21-FLAG, pCA-DDX23-FLAG, or empty vector by linear extrapolation of CT values plotted based on standard curves.; All samples run in triplicate. ***, $P < 0.001$; **, $P < 0.01$; *, $P < 0.05$; ns , $P > 0.05$.

Supplemental Figure2

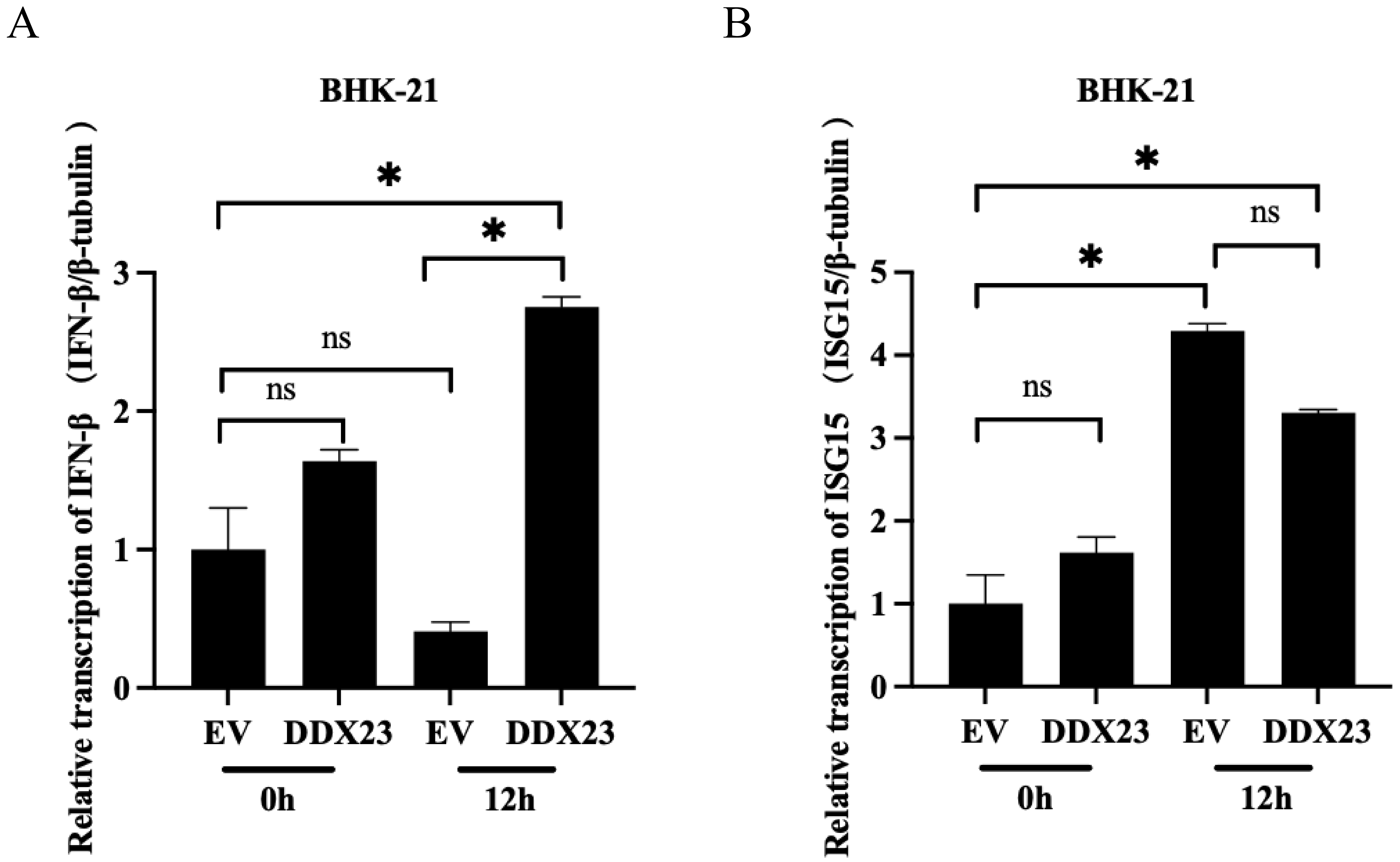

Supplemental Figure2. Effect of SVA infection on IFN- β and ISG15 after overexpression of DDX23 in BHK-21 cells.

(A) BHK-21 cells transfected with pCA-DDX23-FLAG or empty vector were infected with SVA 24 h later, and cells was collected 16 h later for qRT-PCR analysis; (B) BHK-21 cells transfected with pCA-DDX23-FLAG or empty vector were infected with SVA 24 h later, and cells was collected 16 h later for qRT-PCR analysis; All the above Western blot experiments used β-tubulin as the upper sample control. All samples run in triplicate. ***, $P < 0.001$; **, $P < 0.01$; *, $P < 0.05$; ns , $P > 0.05$.

Supplemental Figure 3

A

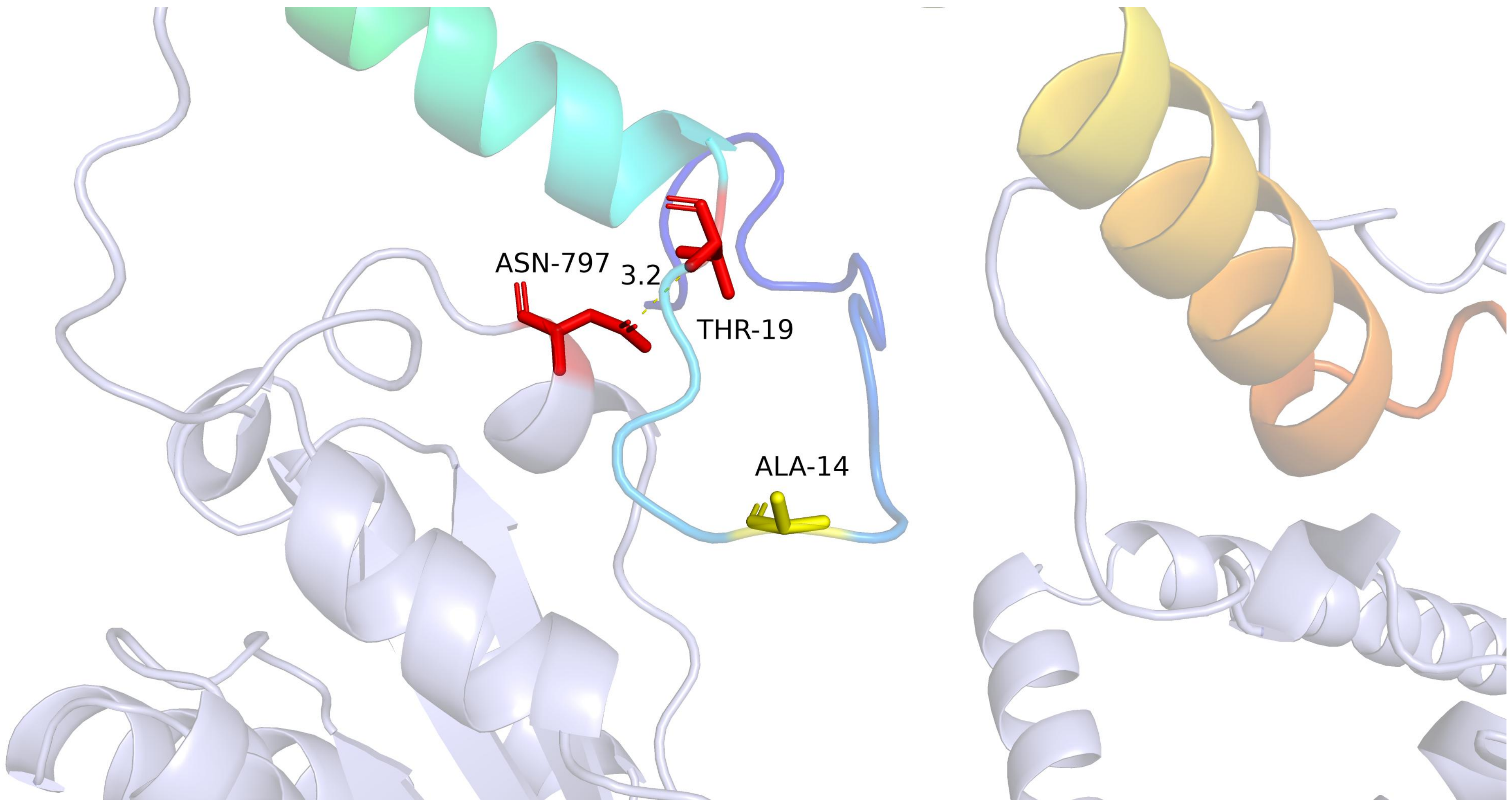

B

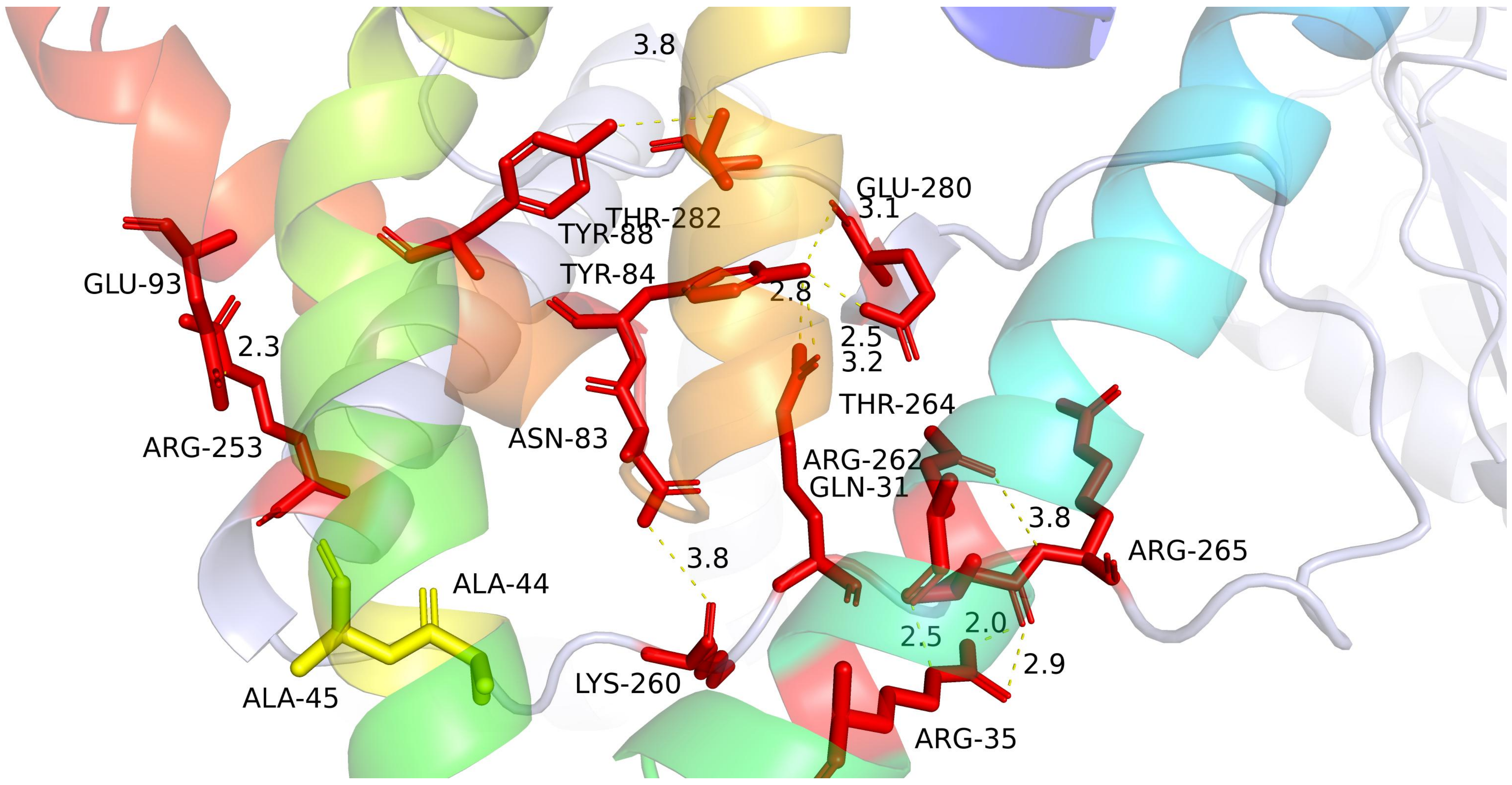

Supplemental Figure 3. Analysis of amino acids involved in the region of interaction between DDX23 and SVA-3A and SVA-2B mutants using 3D modeling

(A) The red color represents the hydrogen bonds that are predicted to form upon interaction, and the yellow color represents the site of the mutant, ALA-14 of SVA-3A did not form a hydrogen bond with DDX23; (B) The red color represents the hydrogen bonds that are predicted to form upon interaction, and the yellow color represents the site of the mutant, ALA-44 and ALA-45 of SVA-2B did not form a hydrogen bond with DDX23
